# Divergent *Pseudomonas aeruginosa* LpxO enzymes perform site-specific lipid A 2-hydroxylation

**DOI:** 10.1128/mbio.02823-23

**Published:** 2023-12-22

**Authors:** Casey E. Hofstaedter, Courtney E. Chandler, Charles M. Met, Joseph J. Gillespie, Janette M. Harro, David R. Goodlett, David A. Rasko, Robert K. Ernst

**Affiliations:** 1 Department of Microbial Pathogenesis, University of Maryland, Baltimore, Baltimore, Maryland, USA; 2 Medical Scientist Training Program, University of Maryland, Baltimore, Baltimore, Maryland, USA; 3 Department of Microbiology and Immunology, University of Maryland Baltimore, Baltimore, Maryland, USA; 4 Departments of Biochemistry and Microbiology, University of Victoria, Victoria, Canada; 5 Institute for Genome Sciences, University of Maryland, Baltimore, Baltimore, Maryland, USA; 6 Center for Pathogen Research, University of Maryland, Baltimore, Baltimore, Maryland, USA; Massachusetts General Hospital, Boston, Massachusetts, USA

**Keywords:** lipid A, *Pseudomonas*, cystic fibrosis, dioxygenases, LPS evolution

## Abstract

*Pseudomonas aeruginosa* can survive in a myriad of environments, partially due to modifications of its lipid A, the membrane anchor of lipopolysaccharide. We previously demonstrated that divergent late acyltransferase paralogs, HtrB1 and HtrB2, add acyloxyacyl laurate to lipid A 2- and 2′-acyl chains, respectively. The genome of *P. aeruginosa* also has genes which encode two dioxygenase enzymes, LpxO1 and LpxO2, that individually hydroxylate a specific secondary laurate. LpxO1 acts on the 2′-acyloxyacyl laurate (added by HtrB2), whereas LpxO2 acts on the 2-acyloxyacyl laurate (added by HtrB1) in a site-specific manner. Furthermore, while both enzyme pairs are evolutionarily linked, phylogenomic analysis suggests the LpxO1/HtrB2 enzyme pair as being of ancestral origin, present throughout the *Pseudomonas* lineage, whereas the LpxO2/HtrB1 enzyme pair likely arose via horizontal gene transfer and has been retained in *P. aeruginosa* over time. Using a murine pulmonary infection model, we showed that both LpxO1 and LpxO2 enzymes are functional *in vivo*, as direct analysis of *in vivo* lipid A structure from bronchoalveolar lavage fluid revealed 2-hydroxylated lipid A. Gene expression analysis reveals increased *lpxO2* but unchanged *lpxO1* expression *in vivo*, suggesting differential regulation of these enzymes during infection. We also demonstrate that loss-of-function mutations arise in *lpxO1* and *lpxO2* during chronic lung infection in people with cystic fibrosis (CF), indicating a potential role for pathogenesis and airway adaptation. Collectively, our study characterizes lipid A 2-hydroxylation during *P. aeruginosa* airway infection that is regulated by two distinct lipid A dioxygenase enzymes.

**IMPORTANCE:**

*Pseudomonas aeruginosa* is an opportunistic pathogen that causes severe infection in hospitalized and chronically ill individuals. During infection, *P. aeruginosa* undergoes adaptive changes to evade host defenses and therapeutic interventions, increasing mortality and morbidity. Lipid A structural alteration is one such change that *P. aeruginosa* isolates undergo during chronic lung infection in CF. Investigating genetic drivers of this lipid A structural variation is crucial in understanding *P. aeruginosa* adaptation during infection. Here, we describe two lipid A dioxygenases with acyl-chain site specificity, each with different evolutionary origins. Further, we show that loss of function in these enzymes occurs in CF clinical isolates, suggesting a potential pathoadaptive phenotype. Studying these bacterial adaptations provides insight into selection pressures of the CF airway on *P. aeruginosa* phenotypes that persist during chronic infection. Understanding these adaptive changes may ultimately provide clinicians better control over bacterial populations during chronic infection.

## INTRODUCTION

As the primary component of the outer leaflet of the outer membrane of most Gram-negative bacteria, lipopolysaccharide (LPS) functions as a permeability barrier and mediator of environmental stressors (1, 2). If recognized by mammalian immune cells, it leads to activation of innate immune responses (i.e., inflammation and secretion of pro-inflammatory cytokines). LPS is composed of three parts: a repeating polysaccharide called O antigen, an oligosaccharide core, and the membrane anchor, lipid A (1). Many Gram-negative pathogens remodel their lipid A in response to environmental stimuli, with these modifications modulating the ability of the toll-like receptor 4 (TLR4)/myeloid differentiation factor 2 (MD-2) receptor complex to trigger an innate immune response (3–5). Lipid A is composed of a diglucosamine backbone that is di-phosphorylated and differentially acylated, depending on bacterial species and environmental conditions (1). Notably, lipid A structure varies among bacterial species, largely due to differences in each bacterium’s repertoire of biosynthetic and modification enzymes (6, 7). Thus, the discovery and description of lipid A modifying enzymes and their impact on lipid A structure are critical for in-depth elucidation of structure-activity relationships that influence immune responses (8).

*Pseudomonas aeruginosa* is a Gram-negative bacterium capable of causing a wide range of infections in plants, mammals, and humans (9). It is found ubiquitously in the environment, where it interacts with other microbes both in soil and water. It is also a common cause of infection for hospitalized or burn patients, or those with compromised immune systems (10, 11). This includes patients with cystic fibrosis (CF), in which *P. aeruginosa* colonizes and infects the airways of over 32% of all patients by early adulthood (12–14). *P. aeruginosa* is afforded environmental flexibility due in large part to an expansive genome containing an enrichment of genes underpinning regulatory pathways and metabolism/transport of organic compounds (15), an elaborate quorum-sensing network (16), and adaptive gene copy number variation (17). However, the larger genome size of *P. aeruginosa* (~6.5 Mb) relative to other extracellular Gram-negative bacteria is not explained by recent gene duplication events, yet rather by numerous small paralogous gene families whose divergent members encode distinct functions (15). The role of these paralogs in ecological versatility is largely unknown and unexplored.

Various Gram-negative bacteria possess LpxO proteins that can produce 2-hydroxylated lipid A structures. The *lpxO* gene was first described in *Salmonella* Typhimurium as encoding a protein that is Fe^2+^ and O_2_ dependent, as well as regulated by the PhoP/Q two-component regulatory system (18, 19). *Klebsiella pneumoniae*, *Acinetobacter baumannii*, and *Bordetella bronchiseptica* also possess a single *lpxO* gene whose product 2-hydroxylates myristate chains in their respective lipid A structures (20–22). Interestingly, some bacteria, such as *Vibrio cholerae*, can produce LpxO-independent hydroxylated lipid A (23). However, these modifications are 3-hydroxylations, instead of the 2-hydroxylations mediated by all LpxO enzymes characterized to date (20–24). Functionally, LpxO has been proposed to mediate resistance to antimicrobial peptides and to contribute to pathogenesis (20–22, 24). The occurrence of two distinct functional *lpxO* genes in *P. aeruginosa* raises questions about their substrate specificities, the origin of their duplicative nature, and the possibility of differing roles in fitness or infection (24).

**Fig 1:**
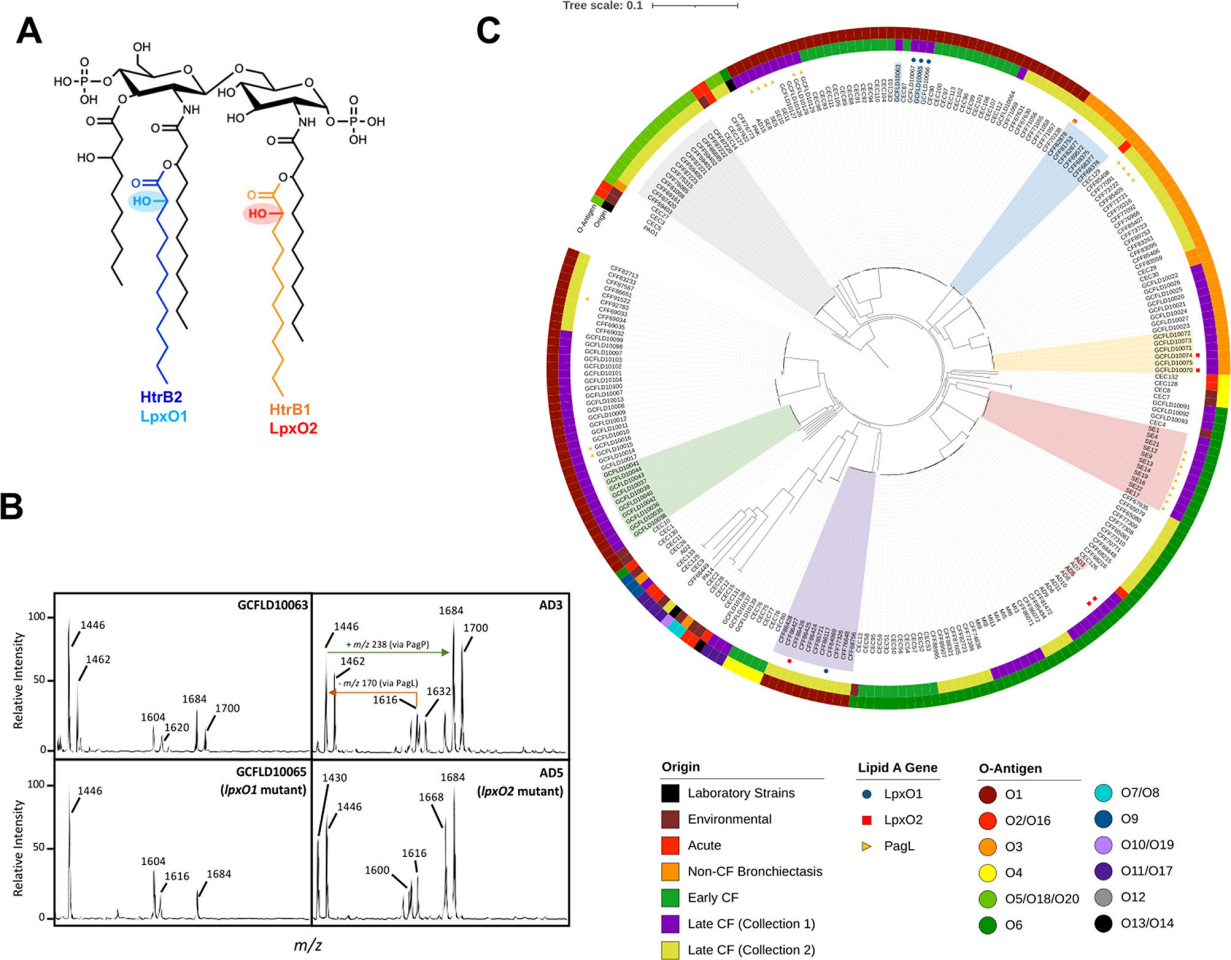
LpxO modifications of *P. aeruginosa* lipid A. (**A**) *P. aeruginosa* lipid A can be doubly 2-hydroxylated on the 2- and 2′-acyloxyacyl laurate chains added by HtrB1 (orange) and HtrB2 (blue), respectively. These modifications are mediated by cognate dioxygenases LpxO2 (red) and LpxO1 (cyan). The size of the lipid A structure presented here is *m*/*z* 1,462. (**B**) matrix-assisted laser desorption/ionization time-of-flight MS spectra of CF isolates grown in lysogenic broth (LB) + 8-µM MgCl_2_. Low Mg^2+^ was used to better assess for lipid A structural variation through increased PhoP/Q activity. Strains from two patients are shown, where GCFLD10063 and GCFLD10065 were isolated from the same patient and AD3 and AD5 were isolated from the same patient. GCFLD10063 (collected at patient age 6.0 years) and AD3 (at patient age 14.85 years) show doubly hydroxylated lipid A as represented by peaks at *m*/*z* values 1,462, 1,632, and 1,700. However, isolates from the same clonal lineage, collected later during infection, lose the doubly hydroxylated lipid A peaks (GCFLD10065 at patient age 13.1 years and AD5 at patient age 15.05 years). These strain names are highlighted on the phylogeny (C). Strain AD3 is annotated with the *m*/*z* differences observed in all strains. (**C**) Phylogeny of 250 *P*. *aeruginosa* genomes was generated based on whole-genome common single-nucleotide polymorphisms (see strain details in Table S1) (25). A total of 179 strains are denoted as late CF and were newly sequenced for this study, whereas *P. aeruginosa* genomes from other sources were also included (26). Sample origin and O antigen are labeled with colored bars. O antigen was determined using *Pseudomonas aeruginosa* serotyper. Isolates with lipid A loss-of-function phenotypes are labeled using the symbols listed under the “Lipid A Gene” heading, and causative mutations are listed in Table S2. Colored wedges denote clonally related isolates from six subjects: Patient 1 (red), Patient 6 (green), Patient 8 (yellow), Patient 15 (gray), Patient 16 (purple), and Patient 22 (blue) (see Table S2 for details) (25–27).

Here, we show that loss-of-function mutations in both *lpxO* genes arise in low-passage clinical *P. aeruginosa* strains, likely to adapt and persist within the CF airway environment. We also demonstrate that LpxO1 and LpxO2 have a unique positional specificity for the 2′-acyloxyacyl laurate (added by HtrB2) and 2-acyloxyacyl laurate (added by HtrB1) chains of *P. aeruginosa* lipid A, respectively (Fig. 1A). Phylogenomic analysis supports that these individual LpxO enzymes arose independently in *Pseudomonas* evolution and codiverged with their cognate HtrB lauryl acyltransferases. Finally, using an intranasal murine infection model, we demonstrate that LpxO1 and LpxO2 are functional *in vivo* by gene expression and mass spectrometric analyses without the requirement for *ex vivo* growth. The *lpxO2* gene expression alone is upregulated *in vivo*, confirming the doubly hydroxylated lipid A phenotype observed using mass spectrometry (MS) and suggesting unique roles for LpxO1 and LpxO2 during airway infection.

## RESULTS

### *LpxO1*/*LpxO2* loss-of-function gene mutations are present in cystic fibrosis *Pseudomonas aeruginosa* strains

*P. aeruginosa* obtained during chronic lung infection in people with cystic fibrosis (pwCF) can have altered lipid A structures when compared to wild-type *P. aeruginosa* lipid A. Interestingly, similar lipid A structural changes are not observed during acute infection; therefore, we hypothesized that lipid A structural modifications arise within the CF airway during chronic infection (28–31). Initially, a cohort of 265 *P*. *aeruginosa* strains obtained from 48 subjects with CF were screened for lipid A structural variation by matrix-assisted laser desorption/ionization time-of-flight (MALDI-TOF) MS (strains listed in Table S1). Altered lipid A structures were observed in 32 strains from 10 subjects. Examples of altered lipid A structure include differences in hydroxylation pattern (∆*m*/*z* 16), acyl-chain number (∆*m*/*z* 170 and *m*/*z* 238), and L-4-aminoarabinose (∆*m*/*z* 131). Lipid A spectra highlighting the change in hydroxylation pattern between early and late CF *P. aeruginosa* strains are shown in Fig. 1B.

Two strains from Patient 7 (GCFLD10063 and GCFLD10065) and two strains from Patient 2 (AD3 and AD5) are presented, where GCFLD10063 and AD3 were collected earlier during chronic lung infection (Fig. 1B, Table S1). All four of these strains demonstrate hexa-acylated (*m*/*z* 1,616) and penta-acylated (*m*/*z* 1,446) lipid A. The penta-acylated lipid A arises as a result of PagL deacylase activity, which specifically removes a 3OH-C10 acyl chain from the hexa-acylated form (32). A second hexa-acylated lipid A form is also present (*m*/*z* 1,684), mediated by PagP, a palmitoyltransferase that adds a C16 acyl chain onto the aforementioned penta-acylated lipid A (33). Both PagL and PagP are present in the outer membrane and exert their effects after lipid A is exported into the outer membrane by the Lpt bridge (32, 33). The *m*/*z* 1,632, *m*/*z* 1,462, and *m*/*z* 1,700 peaks represent lipid A, where both LpxO1 and LpxO2 hydroxylate the secondary acyl chains. Interestingly, for the strains collected later during infection (GCFLD10065 and AD5), the hydroxylation peak pattern is altered, as peaks at *m*/*z* 1,632, *m*/*z* 1,462, and *m*/*z* 1,700 are no longer observed (Fig. 1B), suggesting altered 2-hydroxylation driven by differences in LpxO1 or LpxO2 activity.

To evaluate for genetic drivers of these lipid A phenotypes, we performed whole-genome sequencing (WGS) on all isolates from subjects who demonstrated an altered lipid A phenotype, as described above. Isolates from 12 subjects without lipid A phenotypic differences were also included in this analysis, totaling 179 isolates from 22 CF subjects within the “late CF (Collection 1)” and “late CF (Collection 2)” categories (where the “collection” distinguishes the clinical study from which they were originally obtained). *P. aeruginosa* isolates from environmental sources (“environmental”), acute infection (“acute”), non-CF bronchiectasis (“non-CF bronchiectasis”), and pwCF early in life (<3 years of age) (“early CF”) were also included to assess for genetic relatedness with *P. aeruginosa* from other sources. We aimed to assess the clonality of within-individual longitudinal isolates and whether known lipid A-modifying genes harbor single-nucleotide polymorphism (SNPs) resulting in loss of function, explaining the altered lipid A phenotypes; this was performed using Northern Arizona SNP Pipeline (NASP), a whole-genome SNP analysis pipeline (25). Isolates from each subject were more similar to the other longitudinal isolates from that same subject, as shown by the presence of isolates from the same subject residing on the same clade (Fig. 1C). This finding is consistent with prior genomic studies of longitudinal *P. aeruginosa* isolates in cystic fibrosis (34, 35).

Isolates demonstrating differences in lipid A hydroxylation harbored SNPs in *lpxO1* or *lpxO2* (two and four pwCFs, respectively) and those with differences in lipid A acylation had SNPs in *pagL* (six pwCFs) (Table S2). The observed SNPs suggest truncated proteins, unable to modify lipid A structure (as verified using MALDI-TOF MS). Isolates with altered lipid A phenotypes are designated in Fig. 1C. Interestingly, *lpxO1* and *lpxO2* have not yet been described as pathoadaptive genes in CF (36). Lastly, to assess the hypothesis that mutations in lipid A genes arise in isolates with specific serotypes, we used the *Pseudomonas aeruginosa* serotyper (PAst) program (27). PAst uses WGS data and BLAST to determine the serotype of *P. aeruginosa* based on sequence homology to serotype-specific regions of the genome (27). From this analysis, no apparent relationship between lipid A structural variation and O antigen was observed, suggesting that the observed lipid A structural changes arise independent of O-antigen type (Fig. 1C).

Taken together, *P. aeruginosa* can acquire loss-of-function mutations in *lpxO1* and *lpxO2* during chronic CF lung infection. Interestingly, the presence of two *lpxO* genes in *P. aeruginosa* compared to other highly pathogenic Gram-negative bacteria (i.e., *Klebsiella pneumoniae* and *Acinetobacter baumannii*) is unique; therefore, we further investigated their functions to better understand how they directly impact lipid A structure.

### LpxO1 and LpxO2 have positional specificity

LpxO1 and LpxO2 were previously shown to be capable of hydroxylating *P. aeruginosa* lipid A using MALDI-TOF mass spectrometry (24). However, the exact position of hydroxylation was not explicitly investigated as MALDI-TOF MS (1) cannot distinguish between the exact location of the hydroxyl moieties. Gas chromatography with flame ionization detection (GC-FID) was used to demonstrate the nature of lipid A hydroxylation, as various bacteria have been shown to have 2- or 3-hydroxylated secondary acyl chains (20, 21, 23, 24). GC-FID analysis of wild-type (WT) PAK, a laboratory-adapted strain of *P. aeruginosa*, revealed the presence of both 2- and 3-hydroxylaurate (Fig. S1). This analysis showed that the WT lipid A contained both 2- and 3-hydroxylaurates, whereas a ∆*lpxO1*/*lpxO2* double mutant only showed the presence of 3-hydroxylaurate and a corresponding increase in laurate (Fig. S1). Interestingly, single mutants of ∆*lpxO1* or ∆*lpxO2* showed the presence of 2-hydroxylaurate and suggests that both LpxO1 and LpxO2 are capable of 2-hydroxylation, similar to the canonical LpxO dioxygenase of *Salmonella enterica* (18, 19).

We next tested if the LpxO1 and LpxO2 enzymes could act interchangeably or if they displayed positional specificity. Positional specificity of the LpxO isoforms for lauryl 2-hydroxylation was investigated using tandem mass spectrometry (MS/MS) fragmentation analysis based on the expected lipid A structures. The fragmentation patterns for the penta-acylated, singly hydroxylated ion *m*/*z* 1,446, which is predicted to be 2-hydroxylated at either the 2-acyloxyacyl-laurate or 2′-acyloxyacyl-laurate, are shown in Fig. S2. This ion (*m*/*z* 1,446) from WT lipid A revealed a major cross-ring fragmentation product of *m*/*z* 892, corresponding to laurate at the 2′-acyloxyacyl position (Fig. S2A), as well as a minor cross-ring fragmentation product of *m*/*z* 908 corresponding to 2-hydroxylaurate at the 2′-acyloxyacyl position (Fig. S2B). Deletion of *lpxO1* resulted in *m*/*z* 1,446 with a single major cross-ring fragmentation product of *m*/*z* 892 corresponding to laurate at the 2′-acyloxyacyl position (Fig. S2); thus, *m*/*z* 1,446 from Δ*lpxO1* displayed only a single 2-hydroxylaurate at the 2-acyloxyacyl position. Conversely, deletion of *lpxO2* resulted in *m*/*z* 1,446 with a single major cross-ring fragmentation product of *m*/*z* 908 corresponding to 2-hydroxylaurate at the 2′-acyloxyacyl position (Fig. S2). Thus, *m*/*z* 1,446 from Δ*lpxO2* lipid A displayed 2-hydroxylaurate at the 2*'*-acyloxyacyl position (summarized in Fig. 1A). Together, these results demonstrate that LpxO1 has positional specificity for the 2′-acyloxyacyl laurate, whereas LpxO2 has positional specificity for the 2-acyloxyacyl laurate.

Upon further inspection of *P. aeruginosa* lipid A mass spectra, we also observed differential hydroxylation patterns when LpxO1 or LpxO2 function is absent. Lipid A from *P. aeruginosa* strains lacking LpxO1 function is mono-hydroxylated (no unhydroxylated lipid A peaks at *m*/*z* 1,430 or *m*/*z* 1,600 are present); however, lipid A from LpxO2-deficient *P. aeruginosa* strains demonstrates a mixed lipid A population where there are both unhydroxylated and mono-hydroxylated lipid A species (Fig. 1B). This suggests that 2-acyloxyacyl laurate-2-hydroxylation is dominant, which is mediated by LpxO2, as previously shown (24).

### *lpxO* paralogs arose independently in *Pseudomonas* evolution and have diverged in parallel with their cognate *htrB* genes

**Fig 2:**
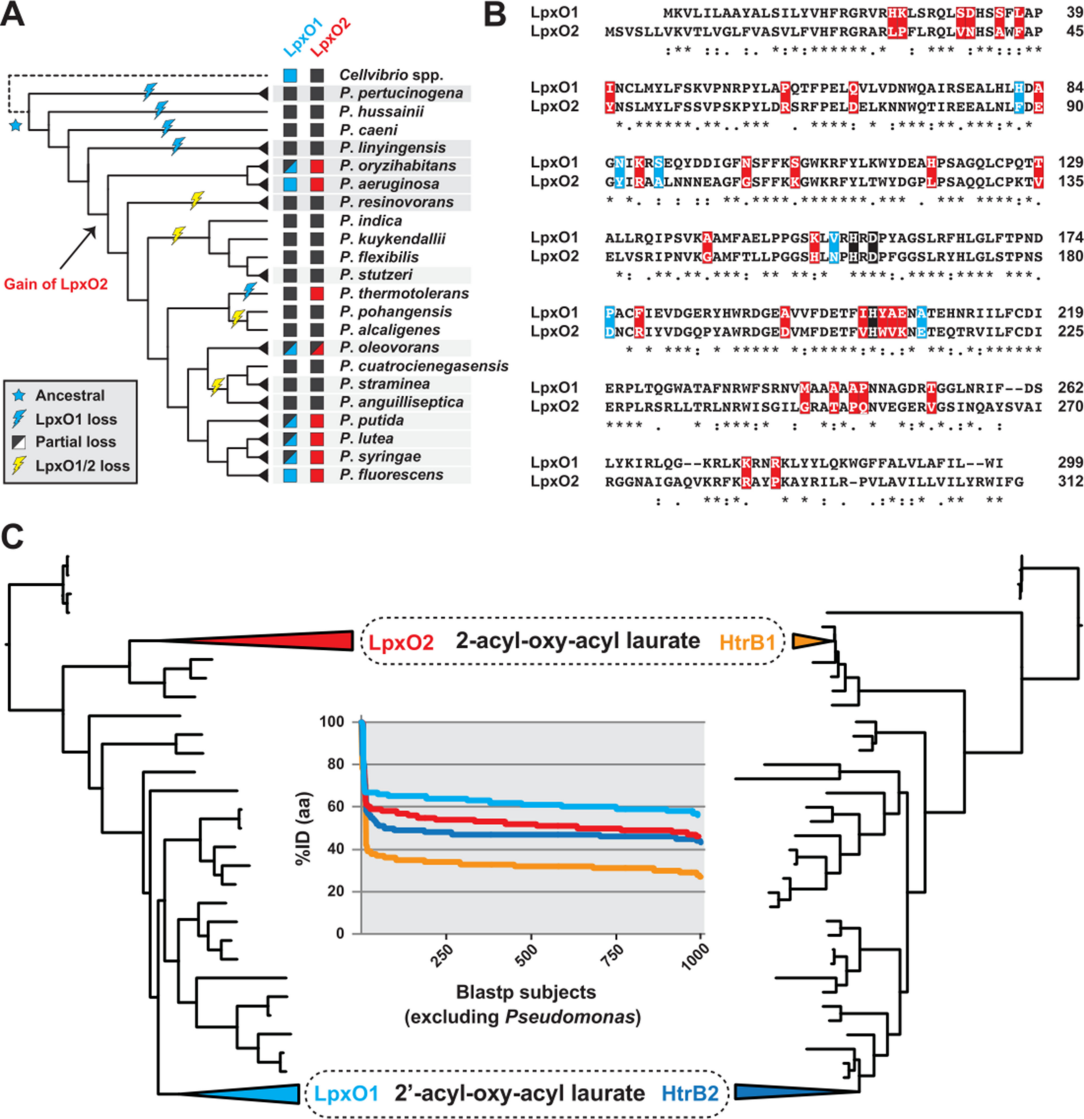
Phylogenomic analysis of *Pseudomonas* 2-hydroxylation of lipid A. (**A**) Phylogenomic analysis of *Pseudomonas* LpxO proteins. Cladogram depicts the *Pseudomonas* phylogeny estimation of Hesse et al. (37), with the recognized groups (containing two or more species) shaded gray. Outgroup *Cellvibrio* spp. (dashed line) carry one LpxO protein with greater amino acid (aa) similarity to LpxO1 (~56% ID) than LpxO2 (~46% ID), allowing for inference of LpxO1 acquisition by vertical inheritance from a pseudomonad ancestor (blue star). LpxO2 is predicted to have arisen later in *Pseudomonas* evolution. Conservation of LpxO1 and LpxO2 sequences is shown to the left of taxon names. *lpxO* losses are inferred over the cladogram with lightning bolts; “partial loss” indicates only some species within groups lacking a particular LpxO enzyme. (**B**) Characterization of *Pseudomonas* LpxO2 divergence. *P. aeruginosa* LpxO1 and LpxO2 are extrapolated from a larger *Pseudomonas* LpxO1/LpxO2 alignment (Fig. S3A); divergent residues conserved within each group are colored blue (LpxO1) or red (LpxO2) and designate the rarer residue found in the analysis of 2096 bacterial LpxO proteins (Fig. S3B and C). LpxO active site residues are colored black. Asterisks (*) denote fully conserved residues; colons (:) indicate conservation between groups of strongly similar properties (scoring >0.5 in the Gonnet PAM 250 matrix); periods (.) indicate conservation between groups of weakly similar properties (scoring ≤0.5 in the Gonnet PAM 250 matrix). Coordinates at the right depict residue numbers within each LpxO protein. (**C**) Phylogeny estimations support independent origins of *Pseudomonas* LpxO1/HtrB2 and LpxO2/HtrB1 gene pairs. Phylograms are simplified (see Fig. S3D and E), and the lineages containing *Pseudomonas* LpxO1, LpxO2, HtrB2, and HtrB1 proteins are collapsed and colored accordingly. Inset: *Pseudomonas* LpxO and HtrB blastp profiles. The top 1,000 blastp subjects (using LpxO1, LpxO2, HtrB2, and HtrB1 as queries in separate searches against the National Center for Biotechnology Information non-redundant protein database, excluding “*Pseudomonas*”) are ranked by decreasing percent amino acid identity.

Having demonstrated that discrete acyltransferase/dioxygenase pairs have positional specificity for 2′-acyloxyacyl laurate (LpxO1/HtrB2) and 2-acyloxyacyl laurate (LpxO2/HtrB1) incorporation into *P. aeruginosa* lipid A, we examined if there was an evolutionary link between these cognate enzymes. While many *Gammaproteobacteria* species carry *lpxO* genes, *P. aeruginosa* is rare in harboring two *lpxO* loci. Phylogenomics-based analysis was used to determine the conservation of *lpxO* duplicate genes across *Pseudomonas* species (Fig. 2A). Evolutionarily distant *Pseudomonas* lineages do not contain *lpxO* genes, with the earliest appearance of both *lpxO1* and *lpxO2* in the *Pseudomonas oryzihabitans* and *P. aeruginosa* groups (Fig. 2A; see arrow). This raises the possibility for acquisition of both *lpxO* genes in these *Pseudomonas* groups. However, we hypothesized that *lpxO1* is derived from an ancestor of the *Pseudomonas* genus, given LpxO1 shares greater similarity to LpxO of pseudomonad relatives (~56% identity on the amino acid level) than does LpxO2 (~46% identity). The gain of *lpxO2* later in *Pseudomonas* evolution, coupled with the patchwork conservation of *lpxO1* and *lpxO2*, indicate differential selection for 2-hydroxylation of lipid A in *Pseudomonas* species.

While *lpxO2* is more conserved than *lpxO1* in *Pseudomonas* species that hydroxylate lipid A (Fig. S3A), LpxO2 proteins are highly divergent from LpxO1 (Fig. 2B) and other bacterial LpxO proteins (Fig. S3C). The lack of conservation of most LpxO2-defining residues in LpxO proteins from other Pseudomonadales species (Fig. S3B and C) raises the possibility that *lpxO2* originated from a non-pseudomonad source. Indeed, phylogeny estimation of select LpxO proteins suggests independent origins of *Pseudomonas lpxO1* and *lpxO2*, with LpxO2 branching outside of Pseudomonadales LpxO proteins (Fig. 2C; Fig. S3D). Phylogeny estimation of HtrB proteins reveals a similar branching pattern for *Pseudomonas* HtrB1 and HtrB2 proteins (Fig. 2C; Fig. S3E), indicating the discrete functional dioxygenase/acyltransferase pairs (LpxO2/HtrB1 and LpxO1/HtrB2) arose independently in *Pseudomonas* evolution, with LpxO2/HtrB1 genes possibly acquired via lateral gene transfer (LGT) after *Pseudomonas* spp. were diverged from other Pseudomonadales. Despite strong functional and phylogenetic links associating these distinct enzyme pairs, all four genes encoding LpxO1, LpxO2, HtrB1, and HtrB2 are encoded at different positions in all *Pseudomonas* genomes (Table S3). This suggests coordinated regulation of cognate dioxygenase/acyltransferase pairs in genomes harboring all four genes.

### Lipid A 2-hydroxylation by LpxO1/LpxO2 does not significantly influence antibiotic susceptibility or signaling through TLR4

The two-component regulatory system PhoP/Q is known to regulate virulence and lipid A-related genes in *P. aeruginosa*, including the lipid A modifying genes *pagL* and *pagP*, both of which are involved in adaptation during CF airway infection (28, 38–40). To determine if *P. aeruginosa lpxO1* and *lpxO2* are likewise regulated by PhoP/Q, lipid A from PAK WT, Δ*phoP*, Δ*phoQ*, and ΔΔ*phoP*/*Q* grown aerobically was extracted and analyzed by MALDI-TOF MS (Fig. S4). The predominant ions in the WT spectra were the same as observed in previous analyses: *m*/*z* 1,446 and *m*/*z* 1,462. As lipid A 2-hydroxylation was observed in Δ*phoP*, Δ*phoQ*, and ΔΔ*phoP*/*Q* genetic backgrounds, these data suggest the PhoP/Q system does not regulate *lpxO* genes in *P. aeruginosa*.

The influence of 2-hydroxylation on lipid A and membrane structure and function is not well understood in *P. aeruginosa*. Loss of *lpxO1*, *lpxO2*, and *lpxO1*/*lpxO2* did not significantly affect bacterial growth at 37°C, as compared to WT PAK (Fig. S5A). However, clinical strains obtained from patients with CF that acquire *lpxO1* loss-of-function mutations *in vivo* have a reduced growth rate when compared to an isolate collected earlier during infection. In one patient, two isolates from the same lineage acquire loss-of-function mutations in *lpxO1* (CFF84969) and *lpxO2* (CFF86426) independently, whereas an earlier isolate obtained from this patient has intact LpxO1/LpxO2 function (CFF77326). The *lpxO1*-deficient strain demonstrates a growth defect, whereas the *lpxO2-*deficient strain does not (Fig. S5F). A similar pattern was observed in isolates from two other patients, where GCFLD10065 (*lpxO1* loss of function) showed a growth defect when compared to an earlier, *lpxO1*/*lpxO2*-competent strain (GCFLD10063) (Fig. S5E), and CFF82878 (*lpxO2* loss of function) showed unchanged growth when compared to an earlier *lpxO1*/*lpxO2*-competent strain (CFF81753) (Fig. S5E). However, when these genes were experimentally deleted from CF isolates obtained from young patients, CEC75 and CEC87 (26, 41), we observe no significant growth alterations. This suggests other compensatory and simultaneous genetic and cellular changes are likely occurring, which lead to selection for loss-of-function mutations in *lpxO1* in the CF lung.

A recent link between lipid A structure and meropenem susceptibility has been described in Enterobacterales (42); therefore, we investigated whether LpxO activity impacts the susceptibility of numerous antibiotics, not just those that target LPS (e.g., polymyxins). Interestingly, we did not observe any significant effects of loss of hydroxylation on antibiotic susceptibility (Table S4). The *lpxO1* and *lpxO2* gene deletions in PAO1, a *P. aeruginosa* laboratory-adapted strain, induce meropenem resistance. Furthermore, for isolates GCFLD10063 and GCFLD10065 (*lpxO1* loss of function)*,* we also observed an increase in resistance to meropenem. However, this pattern was inconsistent with other *P. aeruginosa* strains examined, indicating strain-to-strain variation. Notably, no difference was observed for both *lpxO* mutants in the presence of polymyxin B or colistin (Table S4). When WT PAK was grown in sub-minimum inhibitory concentration (MIC) levels of polymyxin B, the relative intensity of ions correlated to hydroxylated lipid A structure (*m*/*z* 1,446, singly hydroxylated; *m*/*z* 1,462, doubly hydroxylated), which was comparable to WT PAK grown without polymyxin B (Fig. S6). Together, these data suggest that 2-hydroxylation alone does not confer resistance to polymyxin B or other antibiotics evaluated in *P. aeruginosa*. This is unique from what has been observed in *K. pneumoniae* and *A. baumannii*, in which hydroxylation was reported to increase resistance to polymyxin B *in vitro* (20, 21, 43).

**Fig 3:**
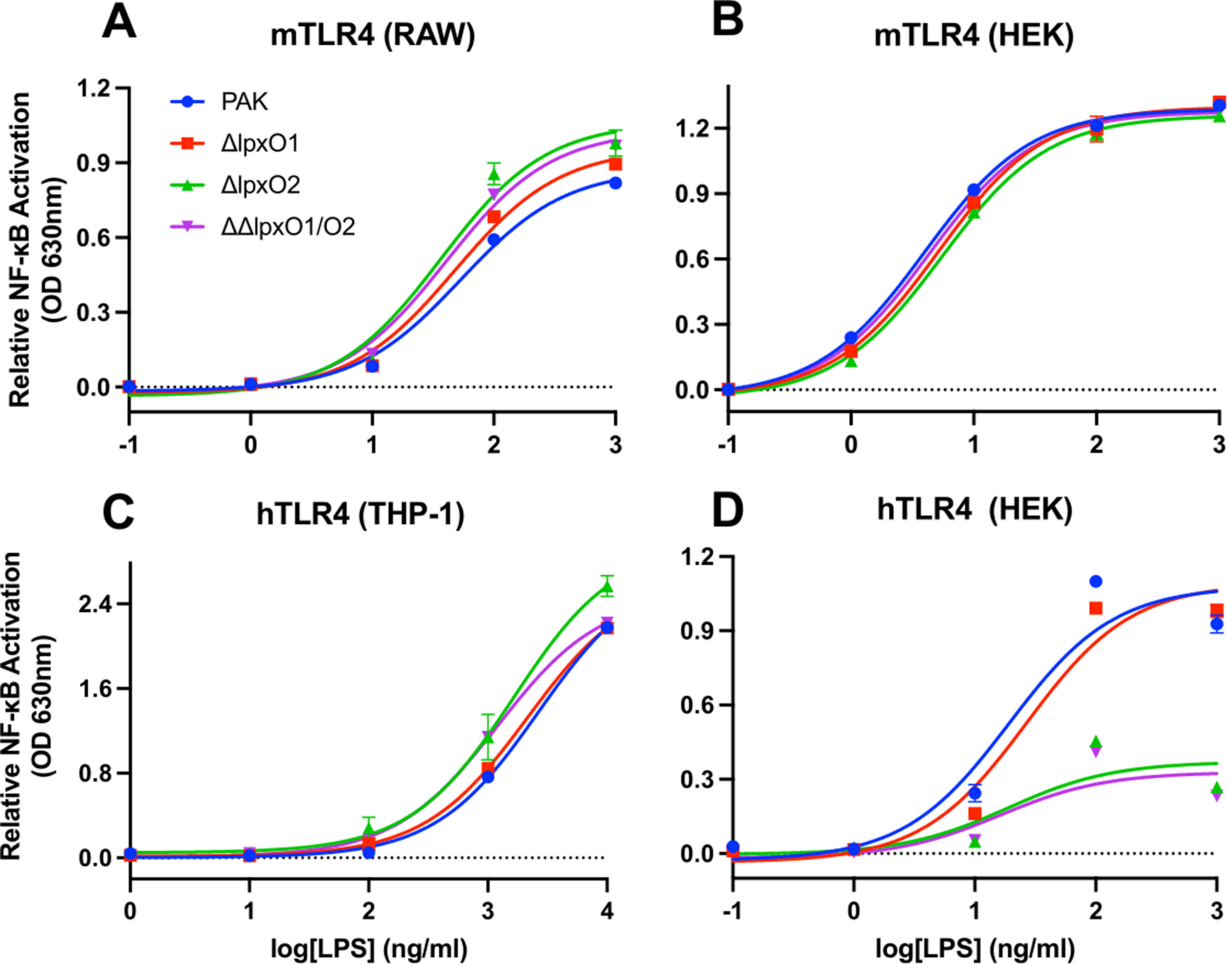
TLR4/MD-2 stimulation by WT and mutant LPS. Cells expressing basal levels of murine TLR4/MD-2 (**A**) and human TLR4/MD-2 (**C**) were stimulated with LPS purified from PAK WT and defined *lpxO* mutants. Cell lines overexpressing murine (**B**) and human (**D**) TLR4/MD-2 were also stimulated. Samples were run in triplicate, but due to limited variation, error bars may not be visible for all samples. Representative data from three independent experiments are shown.

Finally, during infection, lipid A signals through the pattern-recognition receptor TLR4 and coordinating protein MD-2 present on host innate immune cells (44). We used cell expression systems to determine if 2-hydroxylation altered the stimulation potential of lipid A for TLR4, with nuclear factor-kappa B (NF-κB) as the readout for TLR4 signaling. As lipid A is known to differentially activate the murine (m) and human (h) TLR4/MD-2 complexes, cell lines expressing each were used (45, 46). In basal-expressing and overexpressing mTLR4/MD-2 cell lines (RAW and HEK, respectively), loss of 2-hydroxylation had no effect on TLR4 signaling (Fig. 3A and B). In equivalent cell lines expressing hTLR4/MD-2 (THP-1 and HEK, respectively), loss of 2-hydroxylation at the site mediated by LpxO2 decreased stimulation potential when TLR4 is overexpressed (HEK cells) [as determined by a decrease in area under the curve: 1.840 (±0.064) for PAK and 0.6735 (±0.0249) for Δ*lpxO2*] but not when TLR4 is expressed at endogenous levels (THP-1) (Fig. 3C and D). This finding reveals a more nuanced role for *P. aeruginosa* lipid A structure in innate immune recognition.

### LpxO1 and LpxO2 mediate lipid A 2-hydroxylation during murine infection

**Fig 4:**
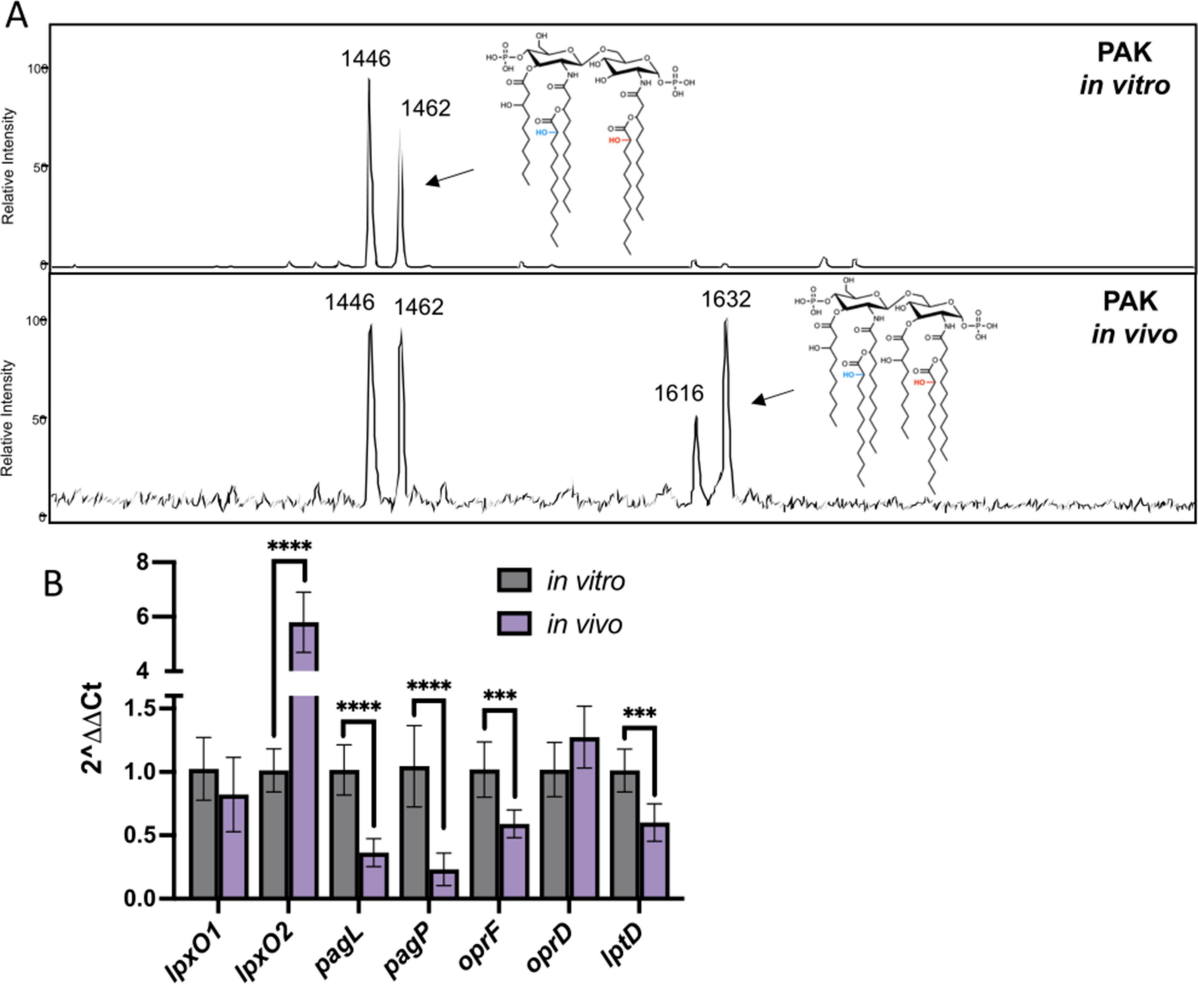
*In vivo P. aeruginosa* lipid A structure and gene expression. (**A**) Lipid A extracted from lung lavage of mice infected (via intranasal route) with *P. aeruginosa* (PAK) revealed singly and doubly 2-hydroxylated lipid A species. Hexa-acylated lipid A species resulting from lack of PagL activity were also observed to be 2-hydroxylated. Hexa-acylated lipid A species were more abundant in *in vivo* extracts compared to *in vitro* extracts (second panel) and were not detected in mock-infected lung lavage extractions (Fig. S7). (**B**) In a similar cohort of animals, whole lungs were isolated; RNA was extracted; and quantitative reverse transcription PCR (qRT-PCR) was performed. *P. aeruginosa* ribosomal protein gene, *rpsL*, was used as a constitutive housekeeping gene for ΔΔC_T_ analysis. Values are relative to PAK grown in lysogenic broth + 1-mM MgCl_2_ to stationary phase. The list of primers used for qRT-PCR are in Table S3. Representative data are shown from one of two independent experiments. ***P < 0.001, ****P < 0.0001.

To date, there are limited data from direct *in vivo* structural analysis of lipid A from Gram-negative bacteria without *ex vivo* culture (21, 47). To analyze *in vivo* lipid A modifications including 2-hydroxylation, we used a model of intranasal pulmonary infection with C57BL/6 mice where lipid A was isolated directly, without culture, from mouse lung lavage after 8 hours of infection. MS analysis of lipid A isolated from lung lavage was markedly different from lipid A extracted from PAK grown in liquid culture *in vitro* (Fig. 4), a pattern that is also consistent with PAO1, another laboratory-adapted *P. aeruginosa* isolate (Fig. S7). The first set of predominant ions in murine lungs were *m*/*z* 1,446 and *m*/z 1,462, corresponding to penta-acylated lipid A with one acyloxyacyl hydroxylaurate and two acyloxyacyl hydroxylaurates, respectively. The next set of predominant ions (*m*/z 1,616 and *m*/*z* 1,632) had approximately equal signal intensity compared to those at *m*/*z* 1,446 and *m*/*z* 1,462, unlike what is observed when *P. aeruginosa* is grown *in vitro* (Fig. 4), although signal intensity is not directly correlated to quantity in MALDI-TOF MS analysis. Ions *m*/*z* 1,616 and *m*/*z* 1,632 correspond to hexa-acylated lipid A with one acyloxyacyl hydroxylaurate and two acyloxyacyl hydroxylaurates, respectively. The hexa-acylation is a result of decreased PagL activity, which removes the 3-hydroxydecanoic acid during lipid A biosynthesis to yield the canonical penta-acylated structure. These results suggest that PagL activity is decreased *in vivo*, yielding a mixture of penta- and hexa-acylated lipid A structures. This observation has not been reported in a mammalian model of *P. aeruginosa* infection to date.

All observed *in vivo* lipid A structures of PAK contained at least one 2-hydroxylation (Fig. 4A). Although MALDI-TOF MS is not quantitative, the relative abundance of doubly 2-hydroxylated lipid A species is increased *in vivo*, as compared to WT PAK grown *in vitro*. Due to the complex nature of the bronchoalveolar lavage fluid, quantitative GC analysis was not possible. This suggests for the first time that both *P. aeruginosa* LpxO enzymes are active during mammalian intranasal infection and, coupled with data from an insect model of infection by Lo Sciuto et al., suggest functional roles for both LpxO enzymes during infection. (24)

To validate the *in vivo* lipid A structure findings, we evaluated *P. aeruginosa* lipid A biosynthesis gene expression *in vivo*. Using the murine model of acute *P. aeruginosa* (PAK) airway infection described above, we harvested lungs for RNA extraction and quantitative reverse transcription PCR (qRT-PCR) analysis of the following *P. aeruginosa* lipid A genes: *pagL*, *pagP*, *lpxO1*, and *lpxO2* (Fig. 4B). Decreased *pagL* expression was observed *in vivo*, validating the mass spectrometry data showing the increased relative intensity of hexa-acylated lipid A (*m*/*z* 1,616 and *m*/*z* 1,632). Interestingly, our data reveal no change in *lpxO1* expression *in vivo* but increased *lpxO2* expression, suggesting differential mechanisms of regulation for each *lpxO* gene (Fig. 4B). *In vivo* gene expression was also examined for the outer membrane beta-barrel responsible for the movement of LPS into the outer membrane (*lptD*) and two outer membrane porins (*oprF*, due to its abundance in the outer membrane, and *oprD,* due to its role as a carbapenem porin). Significant decreases in *lptD* and *oprF* gene expression were observed *in vivo*, suggesting differences in outer membrane protein regulation *in vivo*.

## DISCUSSION

Lipid A molecules from Gram-negative bacteria can be modified to contain 2-hydroxy fatty acids (18–22). However, the genetics, biosynthesis, and function of 2-hydroxy fatty acids are poorly understood. The identification and characterization of two dioxygenases in the *P. aeruginosa* genome suggest that lipid A 2-hydroxylation may play a role in the life cycle or pathogenesis of this bacterium (24). Using a cohort of 179 longitudinal strains from 22 CF patients, we demonstrate that *P. aeruginosa* can acquire loss-of-function mutations in either *lpxO* gene, which may confer survival advantages through antibiotic resistance, altered growth rate, and decreased TLR4 recognition. We establish that LpxO1 (PA4512) and LpxO2 (PA0936) function with positional specificity and are capable of catalyzing the 2-hydroxylation of acyloxyacyl laurate (C12) acyl chains in *P. aeruginosa* lipid A in coordination with the late lipid A acyltransferase enzymes HtrB2 and HtrB1, respectively. However, it remains unknown if LpxO paralogs act sequentially on lipid A in the membrane after the laurate chains have been added or if the laurate substrate is hydroxylated while it is still within the HtrB enzyme before addition to lipid A. Future studies addressing cellular localization of the LpxO and HtrB enzymes, and whether they interact with each other, are needed to better understand their functions.

Phylogenomics analysis indicates that *lpxO* and *htrB* paralogs are present in many *Pseudomonas* species and were acquired independently. We hypothesize vertical descent of LpxO1/HtrB2 genes from a pseudomonad ancestor and probable acquisition of LpxO2/HtrB1 genes via LGT later in *Pseudomonas* evolution. *P. aeruginosa* has many well-documented cases of gene acquisition by LGT, which is partially due to the highly plastic nature of its genome (48). Other Gram-negative bacteria also possess duplications in lipid A acyltransferase genes, including *Klebsiella pneumoniae lpxL*, *Shigella flexneri msbB*, and *Francisella novicida lpxD1* and *lpxD2* (43, 49, 50). The latter paralogs in *Francisella* spp. were also suggested to have occurred via LGT (50). Thus, LGT can be a strong influencer of lipid A structure and associated pathogenesis.

Many environmental and aquatic bacteria have *lpxO* genes or possess *Vibrio*-like hydroxylation modification systems in which a secondary hydroxy-acyltransferase called LpxN transfers 3-hydroxylaurate to the lipid A domain (23). This suggests lipid A hydroxylation may offer an advantage to bacteria in their environmental niches, although more research is needed to identify any specific role. *P. aeruginosa* LpxO enzymes are active during infection in insect and murine models. Our work suggests LpxO2 activity alone impacts signaling through TLR4, whereby LpxO1 activity has a limited role for TLR4 recognition. The impact of 2-hydroxylated lipid A on immune recognition *in vivo* remains unknown (21, 24); however, bacterial medium-chain 3-hydroxy fatty acid metabolites have been shown to activate immune responses in *Arabidopsis* plants (51). Furthermore, during the original work on LpxO in *S. enterica*, it was proposed that liberated 2-hydroxymyristate lipid A chains could function as signaling molecules during intracellular infection (18). It would be interesting to consider the possibility that 2-hydroxylaurate liberated from *P. aeruginosa* lipid A could influence infection via signaling or other roles not traditionally associated with LPS.

Based on this work, lipid A 2-hydroxylation in *P. aeruginosa* mediated by LpxO1 and LpxO2 does not directly impact *in vitro* growth or membrane integrity but may arise with concurrent decreases in growth rate as observed in clinical isolates with naturally acquired *lpxO* loss-of-function mutations (Fig. S5). As in Lo Sciuto et al., we observed minor changes to antibiotic resistance in the presence or absence of lipid A 2-hydroxylation (Table S4) (24). Interestingly, a link between lipid A structure and meropenem susceptibility has been recently described in Enterobacterales (42); however, a direct link between lipid A structure and antimicrobial susceptibility for antibiotics that do not directly target LPS (e.g., carbapenems) is still unclear.

Additionally, more work is needed to fully identify the metabolic benefits that these modifications confer *in vivo* and in environmental niches. It has been speculated that lipid A hydroxylation may provide hydrogen bond donors that function to stabilize lateral interactions within the outer leaflet of the bacterial outer membrane, thus potentially enhancing membrane stability in harsh conditions (52). The ability of *P. aeruginosa* to doubly 2-hydroxylate its lipid A may afford it a selective advantage both during infection and in the environment, where it must overcome competition with various microbial species.

To investigate the *in vivo* lipid A structure of *P. aeruginosa* in an intranasal murine model, which builds on earlier work in *K. pneumoniae* aimed at identifying *in vivo* lipid A structure, we directly analyzed lavage fluid (21). Previous studies, including those conducted by Llobet et al., typically harvest and homogenize infected lungs to analyze lipid A (21). Here, we employed a direct-from-lung MS approach—using a previously described extraction method designed for rapid lipid A extraction—to bronchoalveolar lavage fluid of euthanized mice 8 hours after intranasal infection (53). Our results suggest *P. aeruginosa* lipid A is 2-hydroxylated *in vivo*. Interestingly, the relative abundance of 2-hydroxylated lipid A species seemed to be increased *in vivo* compared to *in vitro*, suggesting a role for both LpxO1 and LpxO2 in pathogenesis. These data provide the first *P. aeruginosa* lipid A spectra directly from murine lavage fluid, which could provide a more rapid means to investigate *in vivo* lipid A structural alterations for a range of Gram-negative bacteria. Interestingly, an increase in *lpxO2* expression with no change in *lpxO1* expression suggests differential regulation *in vivo*, and perhaps unique roles for each LpxO in *P. aeruginosa* infection.

## MATERIALS AND METHODS

### Bacterial strains

*Pseudomonas aeruginosa* strains used in this study are listed in Table S1. Isolates were selected from a bank of *P. aeruginosa* isolates archived in the Cystic Fibrosis Foundation Microbiological Outcomes Advancement Core (*n* = 90, from 10 CF subjects) and from the Ernst Lab Isolate Bank, including strains collected by Samuel Moskowitz at Seattle Children’s Hospital (*n* = 91, from 12 CF subjects). Isolates were identified by the clinical microbiology laboratory following culture on solid tryptic soy agar with 5% sheep blood. Strains were stored in 25% glycerol at −80°C. For aerobic studies, strains were grown in lysogenic broth (LB), incubated at 37°C at 185 rpm, unless otherwise noted.

### DNA extraction

Genomic DNA was isolated from aerobic culture grown at 37°C in lysogeny broth supplemented with 1-mM MgCl_2_ using a GenElute bacterial genomic DNA kit (Sigma-Aldrich, St. Louis, MO). The concentrations of all DNA preparations were determined using a NanoDrop 1000 spectrophotometer (Thermo Fisher Scientific, Waltham, MA). All preparations were stored at −80°C prior to sequencing.

### Whole-genome sequencing

The genomes of all isolates analyzed in this study were sequenced as previously described (41). Sequencing was performed by SeqCenter (www.seqcenter.com). Sample libraries were prepared using the Illumina DNA Prep kit and IDT 10-bp unique dual indexes (UDIs) and sequenced on an Illumina NextSeq 2000, producing 2 × 151 bp reads. All software was used with default values. Raw sequencing reads were filtered to remove contaminating *phiX* reads using BBDuk, one of the BBTools software suite (sourceforge.net/projects/bbmap/). The raw reads were also filtered to remove contaminating Illumina adaptor sequences and quality trimmed using Trimmomatic (version 0.36) (54). The resulting filtered reads were assembled using SPAdes (version 3.13.0) (55). The resulting assemblies were then filtered to contain only contigs longer than 500  bp with a k-mer coverage of ≥5× . Genomes containing more than 500 contigs or an aberrant GC content were removed from further analysis. This Whole Genome Shotgun project has been deposited at GenBank under the Bioproject PRJNA967106.

### Phylogeny estimation

SNPs were identified using NASP (25) with default parameters and PAO1 as a reference (NC_002516_2). SNPs present in all genomes were grouped into a data set for use for phylogeny estimation with iTOL (version 6.7.5).

### *Pseudomonas aeruginosa* O-antigen determination

Following WGS, O-antigen types were identified using the PAst program, as previously described (27). PAst uses WGS data to predict *P. aeruginosa* O antigen using BLAST against serotype-specific genomic regions.

### Recombinant DNA techniques

Strains used in these studies are shown in Table S1. Genetic deletions were generated in the laboratory-adapted strain *P. aeruginosa* PAK using the Gateway Cloning System (Invitrogen, Carlsbad, CA). Clean unmarked deletions of PA0936 (*lpxO2*) and PA4512 (*lpxO1*) were generated as described below. Gateway-compatible vectors pDONR201 with deletions for PA0936 (*lpxO2*) and PA4512 (*lpxO1*) were generated and introduced into *Escherichia coli* DH5α cells by heat shock. Glycerol stocks of pDONR201 plasmid-DH5α cells were struck on plates containing 50-µg/mL kanamycin to select for transformants. Transformants were grown in 5 mL of LB supplemented with 50-µg/mL kanamycin, and pDONR201 plasmid was isolated using a GenElute plasmid miniprep kit (Sigma-Aldrich). The plasmid was then introduced into PAK by electroporation (56). Merodiploids were formed via the integration of the suicide plasmid in a single crossover event. The merodiploid state was resolved in the presence of gentamycin with sucrose counterselection. Deletions were confirmed by PCR sequencing using flanking and intragene primers for each gene of interest. PAKΔ*lpxO1*/*lpxO2* is a double mutant that was generated by deleting *lpxO1* from PAK, then subsequently deleting *lpxO2* from the PAKΔ*lpxO1* strain, as described above.

For PAO1 and CF clinical strains CEC75 and CEC87, genetic deletions were generated using a different method, as previously described, with minor alterations (57). Briefly, PA0936 (*lpxO2*) and PA4512 (*lpxO1*) knock-out constructs were generated in pEX18Gm, with 500 bp upstream and 500 bp downstream of each gene retained in the plasmid for efficient gene deletion (GenScript Biotech, Piscataway, NJ). These plasmids were transformed into *E. coli* S17.1 using electroporation and selected on LB agar plates with gentamicin. These *E. coli* strains were then mated with our *P. aeruginosa* strains of interest. Lastly, merodiploid selection on gentamicin plates, counterselection on sucrose plates, and mutant identification using MALDI-TOF mass spectrometry of purified lipid A (as described below) were performed.

### Small-scale lipid A isolation

Lipid A was isolated from whole cells using an isobutyric acid/ ammonium hydroxide-based extraction procedure as previously described (58). Briefly, cells from approximately 5 mL of culture were centrifuged and the supernatant was removed. Cell pellets were resuspended in 400 µL of 70% isobutyric acid and 1-M ammonium hydroxide 5:3 (vol:vol) and incubated at 100°C for 1 hour. Samples were cooled on ice and centrifuged for 5 minutes at 8,000 × *g*. Supernatants were transferred to a new tube and diluted 1:1 (vol:vol) with endotoxin-free water. Samples were flash-frozen on dry ice and lyophilized overnight. The dried material was washed twice with 1 mL of methanol, and lipid A was extracted in 100 µL of a mixture of chloroform:methanol:water (12:6:1, vol:vol:vol). One microliter of the extract was spotted onto a stainless steel MALDI target plate (Hudson Surface Technology, Fort Lee, NJ) followed by 1 µL of norharmane matrix (Sigma, St. Louis, MO) at a concentration of 10 mg/mL in 2:1 chloroform:methanol (vol:vol). All spots were allowed to air-dry before MALDI-TOF MS analysis.

### Matrix-assisted laser desorption/ionization time-of-flight mass spectrometry

Extracted lipid A was analyzed in reflectron mode on a Bruker microFlex (Bruker Daltonics, Billerica, MA) MALDI-TOF mass spectrometer. Data were acquired in negative ion mode using norharmane as the matrix. The instrument was mass calibrated with an electrospray tuning mix (Agilent, Palo Alto, CA). Data were acquired with flexControl software and processed with flexAnalysis (version 3.4, Bruker Daltonics). All spectra were baseline-smoothed before publication. The resultant spectra were used to estimate the lipid A structures present in each strain based on their predicted or known structures and associated molecular weights.

### Gas chromatography analysis of fatty acid methyl esters

LPS was extracted from 10 mg/mL of lyophilized cell pellets, and fatty acids were converted to fatty acid methyl esters (FAMEs) and analyzed by GC-FID as previously described (59). Briefly, 500 µL of 90% phenol and 500-µL endotoxin-free water was added to 10 mg of lyophilized bacterial cell pellets. Samples were incubated at 70°C for 1 hour with periodic vortexing. Samples were cooled on ice for 5 minutes and centrifuged at 9,391 *× g* for 10 minutes. The top aqueous layer was collected; 500 µL of water was added to the lower organic phase; and samples were incubated again. The process was repeated one additional time for a total of three extractions. All aqueous layers were pooled, and 2 mL of diethyl ether was added. The mixture was vortexed and centrifuged at 2,095 × *g* for 5 min. The lower organic phase was collected, frozen, and lyophilized. The resultant LPS fatty acids were converted to fatty methyl esters in the presence of 10-µg pentadecanoic acid (Sigma) as an internal standard with 2-M methanolic HCl (Alltech, Lexington, KY) at 90°C for 18 hours. Converted fatty methyl esters were then extracted twice with hexane and run on an HP 5890 Series 2 Gas Chromatograph. Retention times from the resultant chromatographs were correlated to fatty acids using GC-FAME standards (Matreya, Pleasant Gap, PA).

### MS/MS fragmentation analysis

Position specificity structural studies were carried out using electrospray ionization linear ion trap Fourier transform ion cyclotron resonance MS as previously described (59).

### Antibiotic susceptibility determination

Antibiotic susceptibility by Kirby-Bauer disk diffusion assay was determined using the standard method described by the Clinical Laboratory Standards Institute (version M07-A10, 10th edition). All antibiotics were purchased from Sigma-Aldrich.

### Growth curve assay

Growth curve measurements (OD_600_) were taken using a Stratus plate reader (Cerillo, Charlottesville, VA). Stationary bacterial cultures were diluted to a McFarland standard of 0.5 in fresh LB to standardized bacterial densities, then further diluted 1:100 in LB. Diluted culture (50 µL) was added to 50-µL fresh LB and added to a 96-well, clear-bottom plate. All samples were run in triplicate, shaking at 180 rpm at 37°C. OD_600_ was recorded every 3 minutes for 20 hours.

### LPS isolation

LPS for cell stimulation studies was purified using a hot phenol/water isolation method as previously described (60). Bacterial pellets from 1-L cultures (grown in LB supplemented with 1-mM MgCl_2_ at 180 rpm, 37°C, for 16 hours) were lyophilized and resuspended in endotoxin-free water to a concentration of 10 mg/mL. A volume of 12.5-mL 90% phenol (Fisher Scientific, Pittsburgh, PA) was added, and the mixture was vortexed and incubated at 65°C for 1 hour. The mixture was cooled on ice, centrifuged at 12,096 × *g* for 30 minutes at room temperature, and the top aqueous phase was collected to a clean tube. An equal volume of endotoxin-free water was added to the organic phase, and the heated incubation and centrifugation were repeated. The aqueous fractions were pooled and dialyzed against Milli-Q purified water for 24 hours to remove any residual phenol. Samples were then lyophilized until dry. The pellet was resuspended to a concentration of 10 mg/mL in endotoxin-free water and treated with DNase (Qiagen, Venlo, Limburg) at 100 µg/mL and RNase A (Qiagen) at 25 µg/mL. Samples were incubated at 37°C for 1 hour in a water bath. Proteinase K (Qiagen) was then added to a final concentration of 100 µg/mL, and the incubation was repeated. An equal volume of water-saturated phenol was then added; the sample was centrifuged as described above; and the aqueous phase was collected and dialyzed against Milli-Q purified water. Samples were then lyophilized. The resultant LPS was further purified to remove contaminating phospholipids by adding 2:1 chloroform:methanol (vol:vol) (61). LPS was further purified by water-saturated phenol extraction followed by 95% ethanol precipitation to remove contaminating lipoproteins (61). Resultant pure LPS was concentrated by lyophilization and stored under nitrogen at −80°C until use. To confirm expected structure, 2 mg of purified LPS was converted to lipid A by mild acid hydrolysis as described previously (62).

### Cell culture and TLR4 stimulation

All mammalian cell cultures were maintained at 37°C with 5% CO_2_. HEK-Blue h TLR4 and HEK-Blue m TLR4 cells (Invitrogen) were cultured in Dulbecco’s Modified Eagle Medium (DMEM) (Gibco, Gaithersburg, MD) supplemented with 10% heat-inactivated fetal bovine serum (FBS) (Sigma-Aldrich), 100-IU/mL penicillin, 100-mg/mL streptomycin, 200-mM L-glutamine, and 1-mM sodium pyruvate. Human THP-1 and murine RAW cells were cultured in Roswell Park Memorial Institute (RPMI) 1640 media (Thermo Fisher Scientific) supplemented with 10% heat-inactivated FBS (Sigma-Aldrich), 100-IU/mL penicillin, 100-mg/mL streptomycin, 200-mM L-glutamine, and 1-mM sodium pyruvate. THP-1 mononuclear cells were cultured with 50-nM vitamin D_3_ (Sigma-Aldrich) for 48 hours to trigger activation and differentiation into macrophage-like cells prior to stimulations. For cell stimulations, purified LPS was reconstituted to a concentration of 1 mg/mL in sterile endotoxin-free water. Multiple aliquots were made and frozen at −20°C until use. Stocks were not used after two freeze-thaw cycles. Stocks were thawed and serially diluted (1:100) in the appropriate cell culture medium before addition to cell culture. Supernatants were collected from cells after 16 hours of stimulation at 37°C with 5% CO_2_. Secreted alkaline phosphatase (SEAP) under control of the NF-κB promoter was used as a colorimetric readout of TLR4/MD-2 stimulation. The production of SEAP reporter was detected using Quanti-Blue (Invitrogen) according to the manufacturer’s instructions. Stimulation data were graphed as the mean ± standard deviation from technical duplicates.

### Phylogenomics analysis

The evolutionary histories of dual LpxO genes were estimated by assessing the occurrence of *lpxO* duplication across the *Pseudomonas* phylogeny. *P. aeruginosa* LpxO1 and LpxO2 were used as queries in blastp searches against the phylogenetic groups proposed by Hesse et al. Searches were performed with composition-based statistics, with no filter used. Default matrix parameters (BLOSUM62) and gap costs (existence: 11, extension: 1) were implemented, with an inclusion threshold of 0.005. Information pertaining to all sequences selected for further analysis are provided in Fig. S3A. One or more (depending on the size of the group) species containing at least one LpxO-encoding gene were selected from each of the major groups (and subgroups of the *P. fluorescens* group).

### Characterizing dual LpxO divergence

Selected *Pseudomonas* LpxO proteins were assessed for conservation relative to other LpxO proteins by assembling a large data set of non-redundant (nr) bacterial LpxO sequences (*n* = 2096), which was retrieved in blastp searches against the National Center for Biotechnology Information (NCBI) nr protein database using both LpxO1 and LpxO2 as queries (using the same blastp search parameters as above) (Table S6). Sequences were aligned using MUSCLE (version 3.8.31) with default parameters (63) (complete methods are described in Fig. S3B and C).

### LpxO and HtrB phylogeny estimations

*Pseudomonas* LpxO and HtrB sequences (Fig. S3A) were merged with other bacterial LpxO and HtrB proteins, using only genomes that carry both *lpxO* and *htrB* genes (to estimate comparable phylogenies). Data sets were constructed via blastp searches against the NCBI “bacteria” nr protein database (as described above) and aligned using MUSCLE (version 3.8.31) (63) with default parameters. Less conserved regions of alignments were masked using Gblocks (version 0.91b), with resulting data sets used in maximum likelihood (ML)-based phylogeny estimations with RAxML (version 8.2.4) (64), which implemented a gamma model of rate heterogeneity and estimation of the proportion of invariable sites. Two evolutionary models were utilized per alignment (WAG and LG), resulting in a total of four ML-based phylogeny estimations. Branch support was assessed with 1,000 pseudoreplications.

### Intranasal infection and lipid A isolation *in vivo*

C57BL/6 mice 6–8 weeks of age were purchased from Jackson Laboratories and housed in a sterile environment. Aerobic cultures of *P. aeruginosa* PAK (a laboratory-adapted strain) was grown at 37°C overnight at 185 rpm. Cultures were diluted to an inoculum size of 1 × 10^8^ CFU in 20 µL of water. Mice were lightly anesthetized with isoflurane, and 10 µL of the inoculum was introduced to each naris. Mice were allowed to recover and monitored every 4 hours for clinical presentation. Eight hours post-infection, mice were euthanized with CO_2_ followed by cervical dislocation. Mouse lungs were lavaged with 200 µL of 100-mM sodium acetate buffer (pH 4.0) under sterile conditions. Lipid A was then extracted from lavage fluid as previously described (53).

### Intranasal infection

Ten 6- to 8-week-old female C57BL/6 mice were infected intranasally with 8.5 × 10^7^ CFU/animal of logarithmic-phase WT *P. aeruginosa* strain PAK. An equal volume of phosphate-buffered saline pH7.4 was used for mock-infected controls. Mice were euthanized after 8 hours, and the right lobe was sterilely excised and immediately placed in 1-mL TRIzol reagent (Invitrogen). Lung tissue immediately underwent RNA extraction.

### RNA extraction

Mouse lungs were mechanically homogenized in TRIzol reagent on ice for 60 seconds, with rest on ice for 30 seconds. This was repeated five times or until the lung was fully homogenized. The standard TRIzol RNA extraction procedure was followed as described by the manufacturer (Invitrogen). Briefly, 200-µL chloroform was added to the TRIzol reagent:lung homogenate mixture; tubes were inverted to mix and sat at room temperature for 2–3 minutes. Samples were centrifuged at 12,000 × *g* for 15 minutes at 4°C. The upper layer was collected, and RNA was purified through the addition of 500-µL isopropanol. Samples were centrifuged; the supernatant discarded; and 1-mL 75% ethanol was added to wash. The samples were centrifuged at 7,500 × *g* for 5 minutes at 4°C. RNA pellets were air-dried for 10 minutes, then resuspended in 50 µL RNase-free water and stored at −80°C. RNA samples underwent DNA digestion using the TURBO DNA-free kit according to manufacturer instructions (Invitrogen).

### cDNA generation and qRT-PCR

The QuantiTect Reverse Transcription Kit (Qiagen) was used for cDNA generation, following manufacturer instructions. qRT-PCR was performed using SsoAdvanced Universal SYBR Green Supermix (Bio-Rad, Hercules, CA) on a CFX Opus 96 Real-Time PCR Instrument (Bio-Rad). Data were analyzed using the ΔΔC_T_ method using *rpsL*, a *P. aeruginosa* ribosomal protein gene, as a control housekeeping gene. Primer sequences are listed in Table S5.

### Data analysis

Where appropriate, data are expressed as means ± standard deviations. Significance was determined using one-way analysis of variance with multiple comparisons in GraphPad Prism (version 7.03) software. A *P* value of <0.05 was considered significant.

## Data Availability

All experimental data will be made available upon request. All sequence data and genome assemblies generated in this study have been submitted to GenBank under BioProject number PRJNA967106 (this study) and PRJNA607994. (26) The individual assembly accession numbers and Illumina sequence read accession numbers are listed in Table S1.

## SUPPLEMENTAL MATERIAL

Figures S1-S6; Tables S3-S5 mbio.02823-23-s0001.pdf Supplemental figures, tables, and references.

Table S1 mbio.02823-23-s0002.xlsx All strains examined in this study.

Table S2 mbio.02823-23-s0003.xlsx Amino-acid altering mutations in lipid A genes for CF clinical *P. aeruginosa* isolates.

Table S6 mbio.02823-23-s0004.xlsx Non-redundant bacterial LpxO sequences from NCBI nr protein database.

ASM does not own the copyrights to Supplemental Material that may be linked to, or accessed through, an article. The authors have granted ASM a non-exclusive, world-wide license to publish the Supplemental Material files. Please contact the corresponding author directly for reuse.
