# Supplementary material for: Divergent Pseudomonas aeruginosa LpxO enzymes perform site-specific lipid A 2-hydroxylation
Source: mBio. 2023 Dec 22;15(2):e02823-23. doi: 10.1128/mbio.02823-23 (PMC10865791; doi:10.1128/mbio.02823-23)
Supplement: Figures S1-S6; Tables S3-S5 — Supplemental figures, tables, and references. [file mbio.02823-23-s0001.pdf]

Supplementary Information for

Divergent *Pseudomonas aeruginosa* LpxO enzymes perform site-specific lipid A 2-hydroxylation.

Casey E. Hofstaedter*[1,2], Courtney E. Chandler*[1], Charles M. Met[1], Joseph J. Gillespie[3], Janette M. Harro[1], David R. Goodlett[4], David A. Rasko[3,5,6], Robert K. Ernst[1,3,6]

*These authors contributed equally to this work

Corresponding:
Robert K. Ernst
Email: rkernst@umaryland.edu

This PDF file includes:

Figures S1 to S7
Tables S1 to S6
SI References

Table S1. Strains examined in this study.

Table S2. SNPs identified in *lpxO1*, *lpxO2*, and *pagL* for *P. aeruginosa* strains examined.

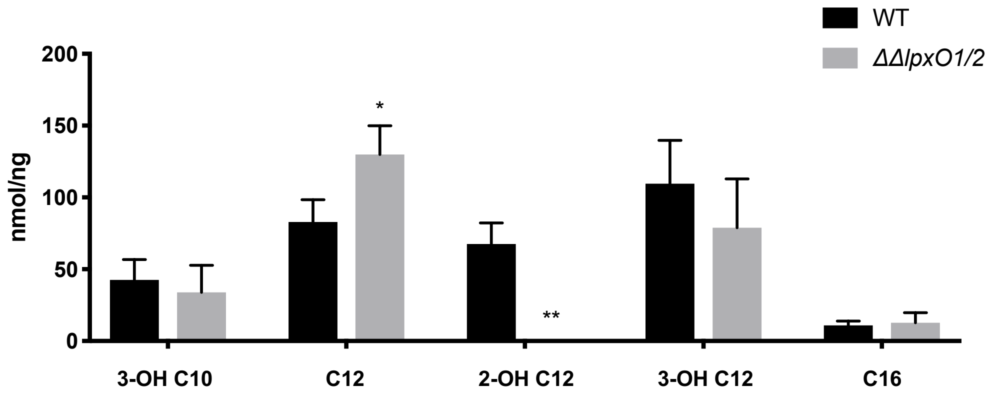

Figure S1. GC-FID analysis of WT and *lpxO1/2* mutant lipid A. Gas chromatography with flame ion detection (GD-FID) was used to analyze fatty acid methyl esters of LPS purified from PAK WT and ΔΔ*lpxO1/2* mutant grown aerobically in LB. For this quantitative structural analysis, the double LpxO1/2 mutant is needed to demonstrate loss of 2-OH C12 within lipid A: presence of either functional LpxO enzyme will result in detectable 2-OH C12. Unpaired t-test was used to compare groups. * indicates $P<0.05$ and ** indicates $P<0.01$.

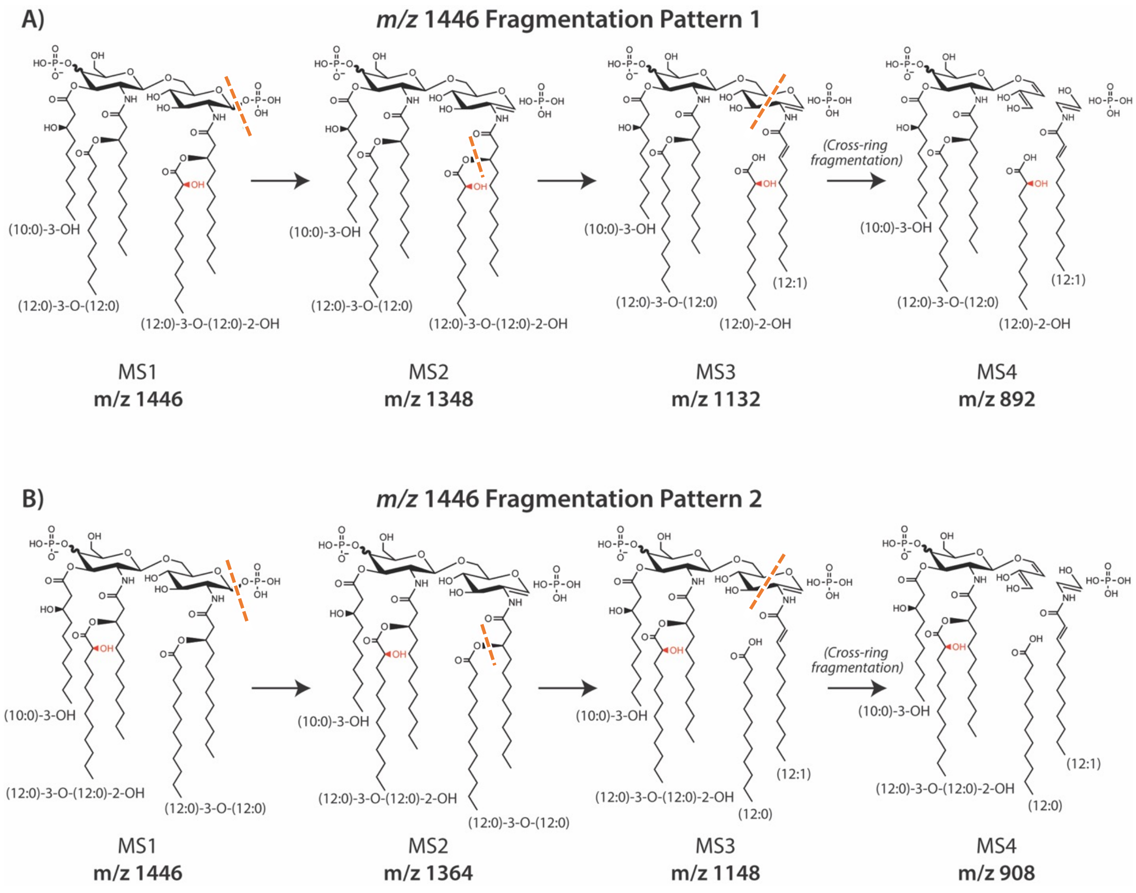

C)

MS1 peak	*m/z* 1446		*m/z* 1616	
MS2 major cross-ring fragment<br>2'-acyl-oxy-acyl substituent	*m/z* 892<br>C12:0	*m/z* 908<br>2OH-C12:0	*m/z* 1062<br>C12:0	*m/z* 1078<br>2OH-C12:0
WT	++	+	+	-
ΔlpxO1	++	-	+	-
ΔlpxO2	-	++	-	+

Figure. S2. (A, B) MS-MS fragmentation patterns of major ions present in *P. aeruginosa* lipid A extracts. Positional specificity of the LpxO1 and LpxO2 enzymes were investigated using MS-MS fragmentation analysis of the major cross-ring fragment (MS4). (C) Summary of MS-MS analysis of lipid A from PAK WT and defined *lpxO* mutants. "-" indicates no ion was observed. "+" indicates the ion was observed. "++" indicates the ion was observed as a dominant ion.

A

Group	Species	LpxO1	LpxO2	HtrB2	HtrB1
OR	*P. oryzihabitans* str. RIT370	KIZ51760	KIZ52847	KIZ51590	KIZ51935
OR	*P. zeshuii* str. KACC 15471	---------------	SHJ29914	SHI34570	SHJ14892
AE	*P. delhiensis* str. CCM 7361	SDK89969	SDK26546	SDJ92114	SDJ70278
PU	*P. coleopterorum* str. LMG 28558	---------------	SEE58563	SED96770	SEE76956
PU	*P. putida* str. KT2440	NP_744571	NP_746679	NP_743891	NP_742233
PU	*P. japonica* str. DSM 22348	SNS60575	SNS57905	SNS80416	SNT02997
LU	*P. lutea* str. LMG 21974	---------------	SER05844	SEQ32464	SEP57137
SY	*P. cichorii* str. ATCC 10857	---------------	SDN30364	SDN31636	SDO55461
SY	*P. syringae* pv. tomato str. NYS-T1	---------------	KGK94113	KGK94142	KGK95198
FL-fr	*P. fragi* str. NRRL B-727	SDU17153	SDU22733	SDU67766	SDU50947
FL-as	*P. asplenii* str. 4A7	PNG44774	PNG44006	PNG42588	PNG40647
FL-ge	*P. gessardii* str. DSM 17152	ONH49242	ONH44142	ONH38551	ONH38840
FL-fl	*P. fluorescens* str. WH6	EFQ63444	EFQ64893	EFQ62138	EFQ66104
FL-pr	*P. protegens* str. Pf-5	AAY92375	AAY90803	AAY93633	AAY95434
FL-ch	*P. chlororaphis* str. O6	EIM15640	EIM14599	EIM17823	EIM18697
FL-co	*P. corrugata* str. RM1-1-4	AOE65241	AOE64481	AOE61672	AOE63100
FL-ko	*P. koreensis* str. D26	AMT89023	AMT87814	AMT88122	AMT86546
FL-je	*P. jessenii* str. LBp-160603	PYC25600	PYC18793	PYC12251	PYC26743
FL-ma	*P. mandelii* str. JR-1	AHZ67365	AHZ69014	AHZ72394	AHZ70516

Figure S3A. Exemplar *Pseudomonas* species used to assess LpxO1 and LpxO2 divergence and estimate LpxO and HtrB phylogenies. After blastp analysis (using queries from *P. aeruginosa* PAO1) to detect genes encoding LpxO1, LpxO2, HtrB2, and HtrB1 (*see Materials and Methods for details*), at least one *Pseudomonas* taxon was selected from the *Pseudomonas* groups and *P. fluorescens* subgroups proposed by Hesse *et al.* (2018). NCBI protein accession numbers are provided. OR, *P. oryzihabitans;* AE, *P. aeruginosa;* PU, *P. putida;* LU, *P. lutea;* SY, *P. syringae;* FL, *P. fluorescens.* Subgroups of *P. fluorescens*: fr, *P. fragi;* ge, *P. gessardii;* fl, *P. fluorescens;* pr, *P. protegens;* ch, *P. chlororaphis;* co, *P. corrugata;* ko, *P. koreensis;* je, *P. jessenii;* ma, *P. mandelii.*

B

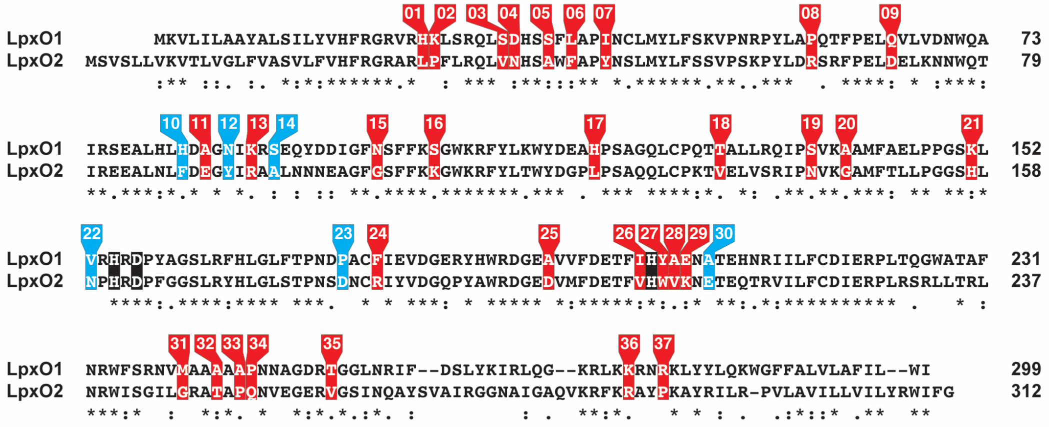

Fig. S3B. Characterization of *Pseudomonas* LpxO1 and LpxO2 divergence. The *P. aeruginosa* LpxO1 and LpxO2 alignment (which summarizes the alignment of all LpxO sequences from panel **A**) has 37 residues highlighted either blue (LpxO1) or red (LpxO2). All highlighted residues indicate divergent residues that are conserved within each group (e.g., highlighted residue 01 is always His in LpxO1 proteins and Leu in LpxO2 proteins). Red (LpxO2) and Blue (LpxO1) highlighting designates the rarer residue found in the larger phylogenomic analysis shown in panel **C**. So, the majority of rarer residues are in LpxO2 proteins (red). LpxO active site residues (His-Asp-His) are colored black. Alignment constructed using MUSCLE v3.8.31[2] with default parameters. Asterisks (*), fully conserved residues; colons (:) indicate conservation between groups of strongly similar properties (scoring > 0.5 in the Gonnet PAM 250 matrix); periods (.) indicate conservation between groups of weakly similar properties (scoring =< 0.5 in the Gonnet PAM 250 matrix). Coordinates at right depict residue numbers within each LpxO protein.

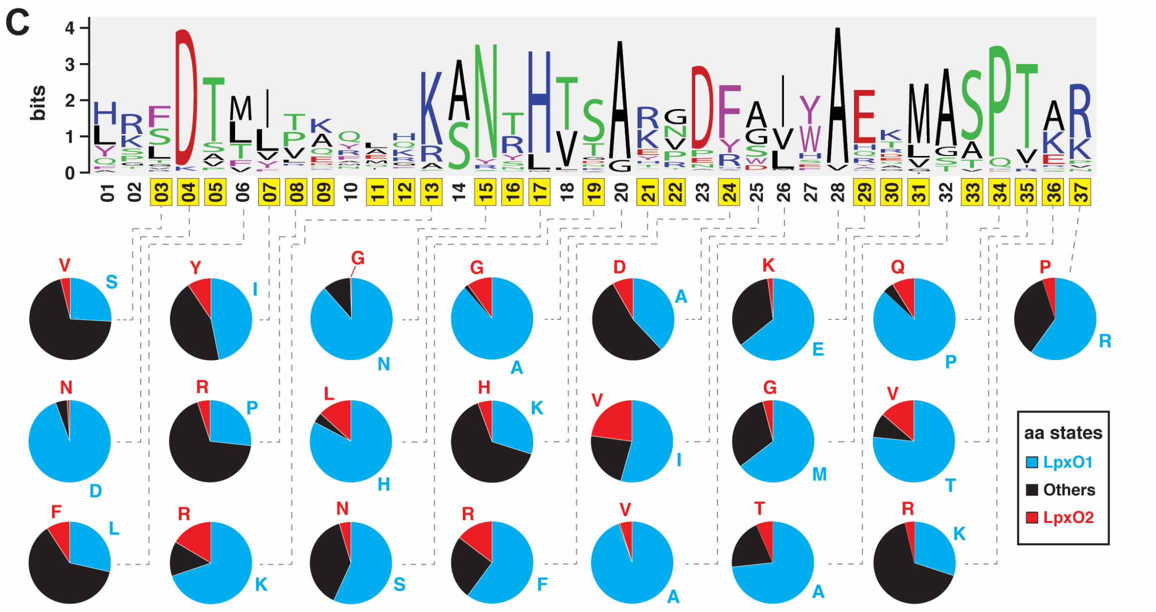

Figure S3C. Large-scale phylogenomics-based assessment of the residues that are uniquely conserved in *Pseudomonas* LpxO1 or LpxO2 sequences. Non-redundant bacterial LpxO sequences (n = 2096) were retrieved in blastp searches against the NCBI nr protein database using both *P. aeruginosa* LpxO1 and LpxO2 as queries (*see Materials and Methods for blastp parameters*), with subjects compiled and aligned using MUSCLE v3.8.31[2] with default parameters. The sequence logo (generated with WebLogo[3]) illustrates the relative conservation of the 37 highlighted residues in panel B across the alignment of these 2096 diverse LpxO proteins. Pie charts (generated only for residues where the LpxO1 amino acid (aa) state is present in 25% of the proteins (524 or more sequences) and at a two-fold greater frequency than the LpxO2 aa state) illustrate the breakdown of the LpxO1, LpxO2 and other (non-*Pseudomonas*) LpxO aa states across the alignment. Yellow highlighting depicts 24 residues where only less than five non-*Pseudomonas* species of Pseudomonadales were found to contain the LpxO2-defining aa state (illustrating the uniqueness of the LpxO2 aa states and probable origin of LpxO2 from outside of Pseudomonadales).

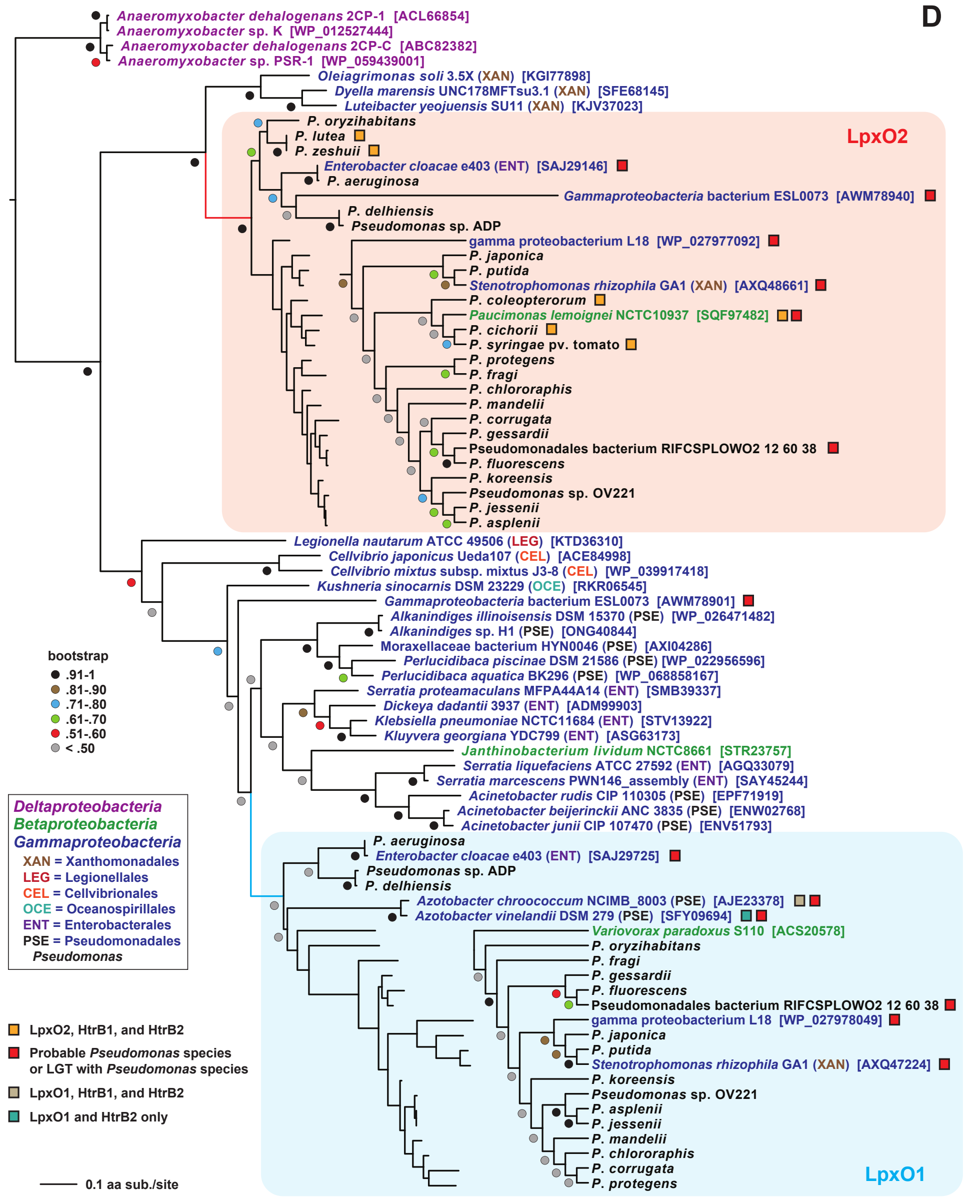
D
Anaeromyxobacter dehalogenans 2CP-1 [ACL66854]
Anaeromyxobacter sp. K [WP_012527444]
Anaeromyxobacter dehalogenans 2CP-C [ABC82382]
Anaeromyxobacter sp. PSR-1 [WP_059439001]
Oleiagrimonas soli 3.5X (XAN) [KGI77898]
Dyella marensis UNC178MFTsu3.1 (XAN) [SFE68145]
Luteibacter yeojuensis SU11 (XAN) [KJV37023]
LpxO2
P. oryzihabitans
P. lutea
P. zeshuii
Enterobacter cloacae e403 (ENT) [SAJ29146]
P. aeruginosa
Gammaproteobacteria bacterium ESL0073 [AWM78940]
P. delhiensis
Pseudomonas sp. ADP
gamma proteobacterium L18 [WP_027977092]
P. japonica
P. putida
Stenotrophomonas rhizophila GA1 (XAN) [AXQ48661]
P. coleopterorum
Paucimonas lemoignei NCTC10937 [SQF97482]
P. cichorii
P. syringae pv. tomato
P. protegens
P. fragi
P. chlororaphis
P. mandelii
P. corrugata
P. gessardii
Pseudomonadales bacterium RIFCSPLOWO2 12 60 38
P. fluorescens
P. koreensis
Pseudomonas sp. OV221
P. jessenii
P. asplenii
Legionella nautarum ATCC 49506 (LEG) [KTD36310]
Cellvibrio japonicus Ueda107 (CEL) [ACE84998]
Cellvibrio mixtus subsp. mixtus J3-8 (CEL) [WP_039917418]
Kushneria sinocarnis DSM 23229 (OCE) [RKR06545]
Gammaproteobacteria bacterium ESL0073 [AWM78901]
Alkanindiges illinoisensis DSM 15370 (PSE) [WP_026471482]
Alkanindiges sp. H1 (PSE) [ONG40844]
Moraxellaceae bacterium HYN0046 (PSE) [AXI04286]
Perlucidibaca piscinae DSM 21586 (PSE) [WP_022956596]
Perlucidibaca aquatica BK296 (PSE) [WP_068858167]
Serratia proteamaculans MFPA44A14 (ENT) [SMB39337]
Dickeya dadantii 3937 (ENT) [ADM99903]
Klebsiella pneumoniae NCTC11684 (ENT) [STV13922]
Kluyvera georgiana YDC799 (ENT) [ASG63173]
Janthinobacterium lividum NCTC8661 [STR23757]
Serratia liquefaciens ATCC 27592 (ENT) [AGQ33079]
Serratia marcescens PWN146_assembly (ENT) [SAY45244]
Acinetobacter rudis CIP 110305 (PSE) [EPF71919]
Acinetobacter beijerinckii ANC 3835 (PSE) [ENW02768]
Acinetobacter junii CIP 107470 (PSE) [ENV51793]
P. aeruginosa
Enterobacter cloacae e403 (ENT) [SAJ29725]
Pseudomonas sp. ADP
P. delhiensis
Azotobacter chroococcum NCIMB_8003 (PSE) [AJE23378]
Azotobacter vinelandii DSM 279 (PSE) [SFY09694]
Variovorax paradoxus S110 [ACS20578]
P. oryzihabitans
P. fragi
P. gessardii
P. fluorescens
Pseudomonadales bacterium RIFCSPLOWO2 12 60 38
gamma proteobacterium L18 [WP_027978049]
P. japonica
P. putida
Stenotrophomonas rhizophila GA1 (XAN) [AXQ47224]
P. koreensis
Pseudomonas sp. OV221
P. asplenii
P. jessenii
P. mandelii
P. chlororaphis
P. corrugata
P. protegens
LpxO1
bootstrap
.91-1
.81-.90
.71-.80
.61-.70
.51-.60
< .50
Deltaproteobacteria
Betaproteobacteria
Gammaproteobacteria
XAN = Xanthomonadales
LEG = Legionellales
CEL = Cellvibrionales
OCE = Oceanospirillales
ENT = Enterobacterales
PSE = Pseudomonadales
Pseudomonas
LpxO2, HtrB1, and HtrB2
Probable Pseudomonas species or LGT with Pseudomonas species
LpxO1, HtrB1, and HtrB2
LpxO1 and HtrB2 only
0.1 aa sub./site

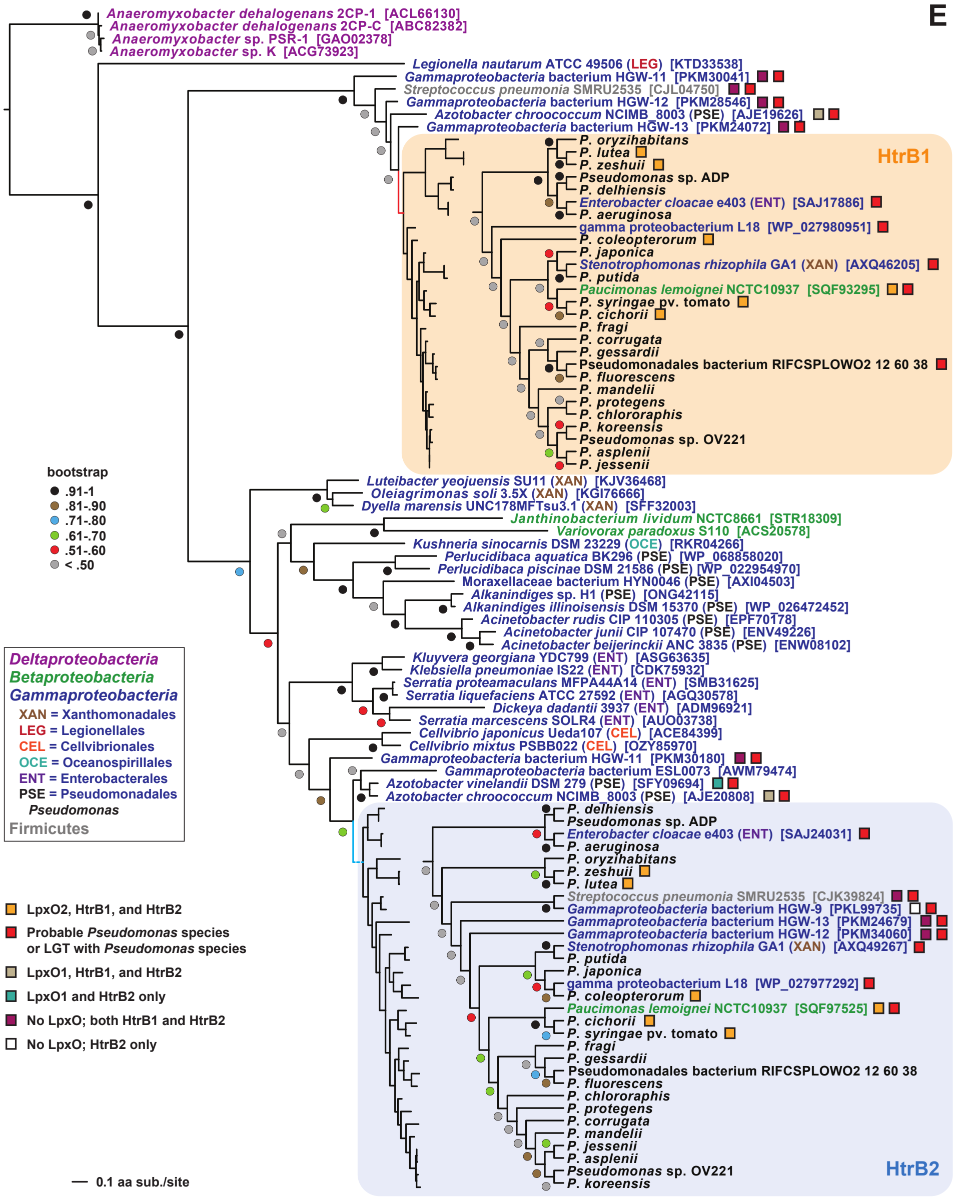

Figure S3D-E. Phylogeny estimations for LpxO and HtrB proteins. Datasets utilized to determine the evolutionary history of *Pseudomonas* LpxO and HtrB proteins were constructed using genomes that carry both *lpxO* and *htrB* genes (note: nine assemblies lacking LpxO genes were included in the HtrB phylogeny estimation since these sequences are very similar to *Pseudomonas* counterparts). This entailed combining the sequences from panel A with taxa selected from blastp searches against the NCBI ‘bacteria’ nr protein database. LpxO and HtrB datasets were aligned using MUSCLE v3.8.31[2] with default parameters. Alignments were masked using Gblocks 0.91b, with resulting alignments used in maximum likelihood (ML)-based phylogeny estimations with RAxML v8.2.4[4], which implemented a gamma model of rate heterogeneity and estimation of the proportion of invariable sites. Two evolutionary models were utilized per alignment (WAG and LG), resulting in a total of four ML-based phylogeny

estimations (LG-based trees shown here are highly corroborated by WAG-based estimations). Branch support was assessed with 1,000 pseudoreplications. (D) LpxO phylogeny. The alignment (79 sequences, 365 positions) was trimmed to 266 positions. The final LogLikelihood was -11669.644136. (E) HtrB phylogeny. The alignment (94 sequences, 366 positions) was trimmed to 244 positions. The final LogLikelihood was -15110.411417.

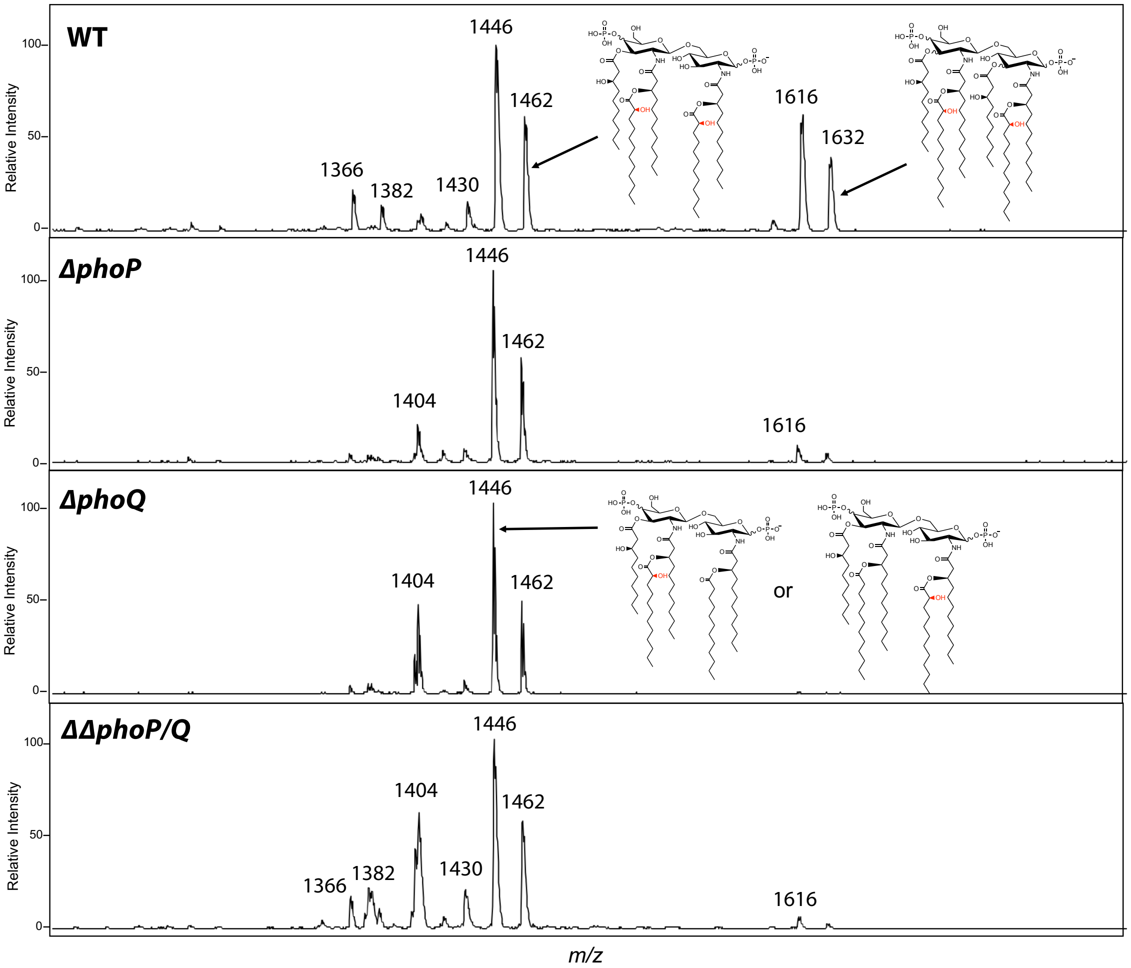

Figure S4. MALDI-TOF MS analysis of WT (PAK) and defined *phoP/Q* mutant lipid A structures extracted from aerobic cultures. *m/z* 1366, 1382, 1446, and 1462 correspond to penta-acylated lipid A structures with one or two points of 2-hydroxylation. *m/z* 1616 and 1632 correspond to hexa-acylated lipid A structure with one or two points of 2-hydroxylation, respectively. Bacterial strains were grown in low magnesium (8μM $MgCl_2$) conditions to induce PhoP/Q activity, resulting in increased hexa-acylated lipid A for wild-type PAK.

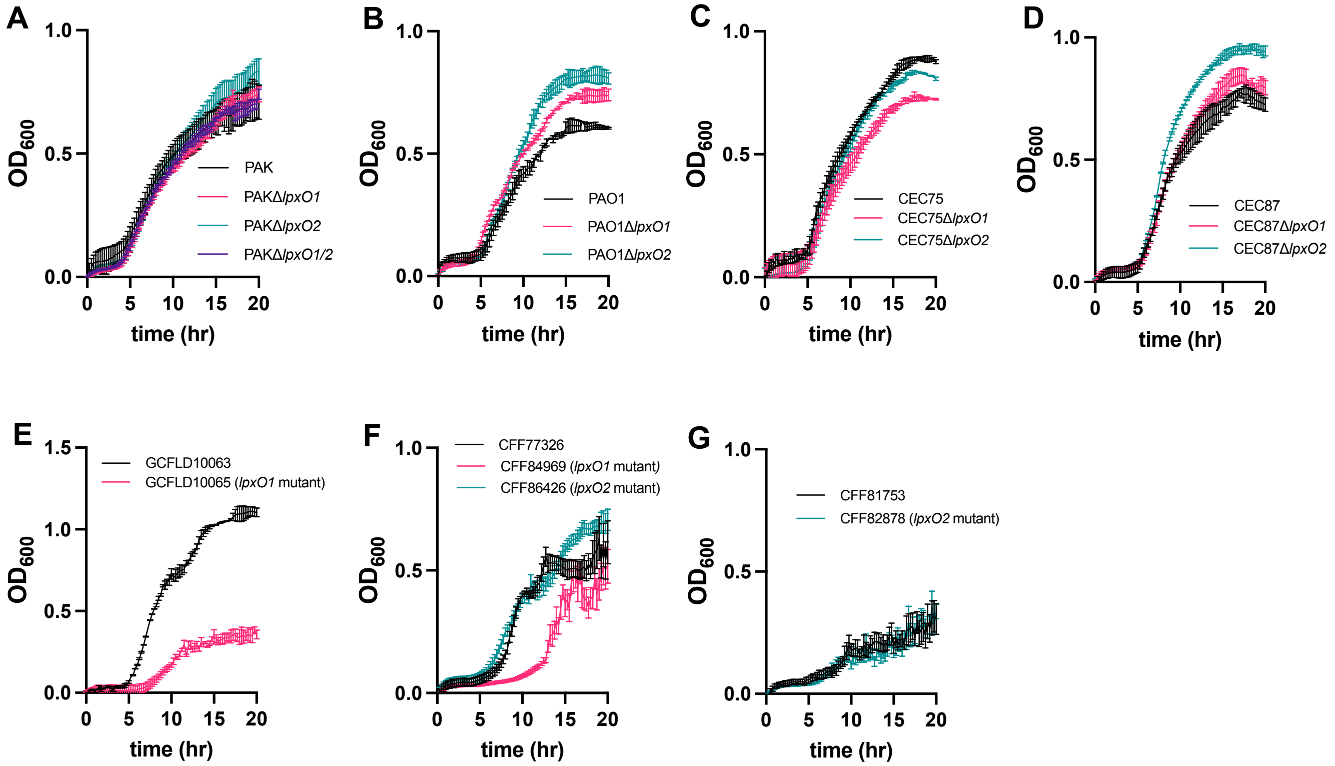

Figure S5. *In vitro* growth curves for PAK, PAO1 and clinical *lpxO* mutant strains. Bacteria were grown in LB at 37C, shaking at 180rpm for 20 hours. OD_{600} measurements were taken every 15 minutes using a Stratus plate reader (Cerillo, Charlottesville, VA). Laboratory-adapted strains PAK and PAO1 with their experimentally-introduced gene deletions are shown in panels (A) and (B), and early CF strains from two patients with experimentally-introduced *lpxO* gene deletions are shown in panels (C) and (D). Panels (E-G) depict three CF subjects who have isolates with *lpxO*-deficient strains. Strains with loss-of-function mutations in one *lpxO* gene are labeled in parentheses. An earlier isolate from each patient was chosen as a *lpxO*-competent control. Strain 77326 has clonal decedents that acquired loss-of-function mutations in *lpxO1* and *lpxO2* independently. Each strain was run in triplicate. Error bars represent standard-deviation of replicates.

Table S3. Gene location in *P. aeruginosa* genome (PAO1 and PAK). Gene location was determined as previously described, using *Pseudomonas aeruginosa* PAO1, assembly accession GCF_000006765.1, and *Pseudomonas aeruginosa* PAK, assembly accession LR657304.1.[5,6]

Strain	Gene	PA locus	genome location (nucleotide number)
PAO1	*lpxO1*	PA4512	5049769 - 5050668
PAO1	*lpxO2*	PA0936	1026274 - 1027212
PAO1	*htrB1*	PA0011	14235 - 15122
PAO1	*htrB2*	PA3242	3629667 - 3630605
PAK	*lpxO1*	PAKAF_04783	5158307 - 5159206
PAK	*lpxO2*	PAKAF_04252	4565727 - 4566665
PAK	*htrB1*	PAKAF_00011	13843 - 14628
PAK	*htrB2*	PAKAF_01743	1868135 - 1869073

Table S4. Antibiotic resistance of *lpxO* mutants. Using Kirby-Bauer antibiotic disk diffusion assay, bacterial clearance diameter (in millimeters) is given to determine resistance for each antibiotic tested, as previously described.[7] Average values are shown from three unique experiments, where standard deviations are in the range of 1-10% for all values. Antibiotic abbreviation and dosage (in micrograms per disc) are described in the "ID" column.

Antibiotic Class	Antibiotic	ID	PAK	PAKΔ*lpxO1*	PAKΔ*lpxO2*	PAKΔ*lpxO1/2*
	Rifampicin	RD-5	0	0	0	0
Beta-lactams	Ampicillin/Sulbactam	SAM-20	0	0	0	0
	Aztreonam	ATM-30	32	30	33	32
	Meropenem	MEM-10	21	22	24	24
	Ceftazidime	CAZ-30	11	0	13	12
Macrolide	Erythromycin	E-15	11	11	11	11
Tetracycline	Tetracycline	TE-30	0	10	12	11
	Trimethoprim	W-5	0	0	0	0
Aminoglycosides	Tobramycin	TOB-10	25	26	27	25
Polymyxins	Polymyxin B	PB-300	15	15	15	15
	Colistin	CT-10	16	15	15	15

Antibiotic Class	Antibiotic	ID	PAO1	PAO1Δ*lpxO1*	PAO1Δ*lpxO2*
	Rifampicin	RD-5	0	0	0
Beta-lactams	Ampicillin/Sulbactam	SAM-20	0	0	0
	Aztreonam	ATM-30	31	30	28
	Meropenem	MEM-10	31	0	0
	Ceftazidime	CAZ-30	0	0	16
Macrolide	Erythromycin	E-15	12	14	12
Tetracycline	Tetracycline	TE-30	10	13	11
	Trimethoprim	W-5	0	0	0
Aminoglycosides	Tobramycin	TOB-10	25	26	25
Polymyxins	Polymyxin B	PB-300	13	14	14
	Colistin	CT-10	14	14	15

KEY
**Resistant**
**Intermediate**
**Susceptible**

Antibiotic Class	Antibiotic	ID	CEC75	CEC75Δ*lpxO1*	CEC75Δ*lpxO2*	CEC87	CEC87Δ*lpxO1*	CEC87Δ*lpxO2*
	Rifampicin	RD-5	0	0	0	0	0	0
Beta-lactams	Ampicillin/Sulbactam	SAM-20	0	0	0	0	0	0
	Aztreonam	ATM-30	23	23	25	25	28	24
	Meropenem	MEM-10	12	0	0	0	0	0
	Ceftazidime	CAZ-30	0	0	0	0	0	12
Macrolide	Erythromycin	E-15	11	12	12	14	15	10
Tetracycline	Tetracycline	TE-30	12	10	12	13	11	12
	Trimethoprim	W-5	0	0	0	0	0	0
Aminoglycosides	Tobramycin	TOB-10	22	22	23	23	23	20
Polymyxins	Polymyxin B	PB-300	14	14	14	15	19	22
	Colistin	CT-10	14	14	15	16	15	14

Antibiotic Class	Antibiotic	ID	81753	82878 (Δ*lpxO1*)	10063	10065 (Δ*lpxO1*)	77326	84969 (Δ*lpxO1*)	86426 (Δ*lpxO2*)
	Rifampicin	RD-5	0	0	0	0	0	0	0
Beta-lactams	Ampicillin/Sulbactam	SAM-20	0	0	0	0	26	0	13
	Aztreonam	ATM-30	25	26	27	0	39	34	44
	Meropenem	MEM-10	15	25	25	0	45	46	40
	Ceftazidime	CAZ-30	0	0	11	0	0	0	0
Macrolide	Erythromycin	E-15	0	0	0	0	0	0	0
Tetracycline	Tetracycline	TE-30	13	0	10	13	0	11	12
	Trimethoprim	W-5	0	0	0	0	0	0	0
Aminoglycosides	Tobramycin	TOB-10	12	12	19	17	19	19	19
Polymyxins	Polymyxin B	PB-300	16	19	15	17	15	16	16
	Colistin	CT-10	18	19	14	18	16	18	18

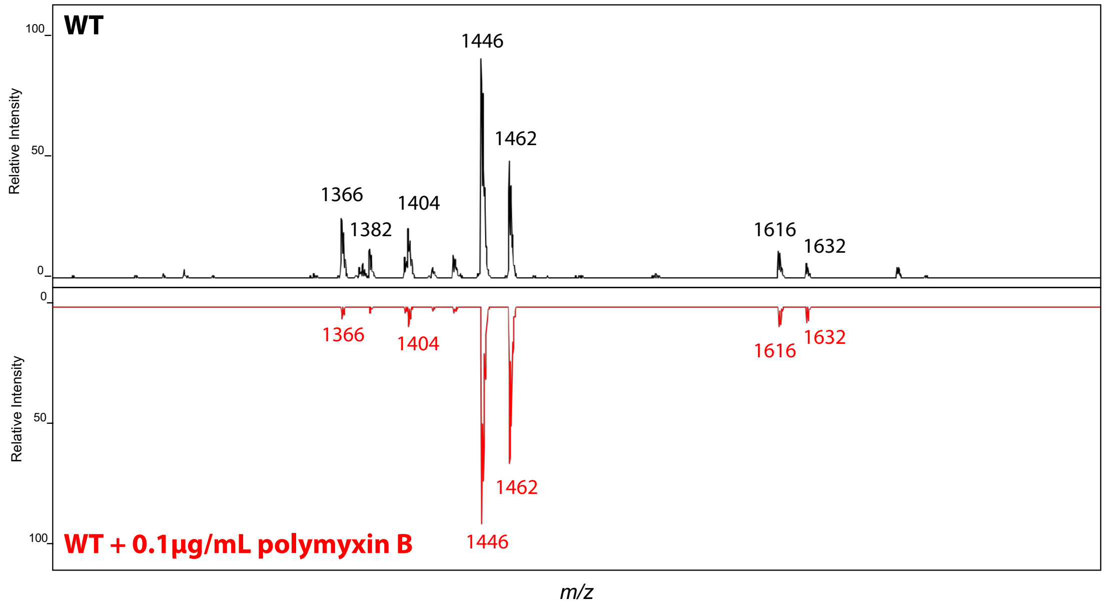

Figure S6. Mirror plot of MALDI-TOF MS analysis of lipid A extracted from PAK WT grown in LB media (top panel, black) or LB media supplemented with sub-MIC levels (0.1mg/mL) polymyxin B (bottom panel, red).[8] Polymyxin B did not appear to alter the relative abundance ions representative of 2-hydroxylated lipid A species.

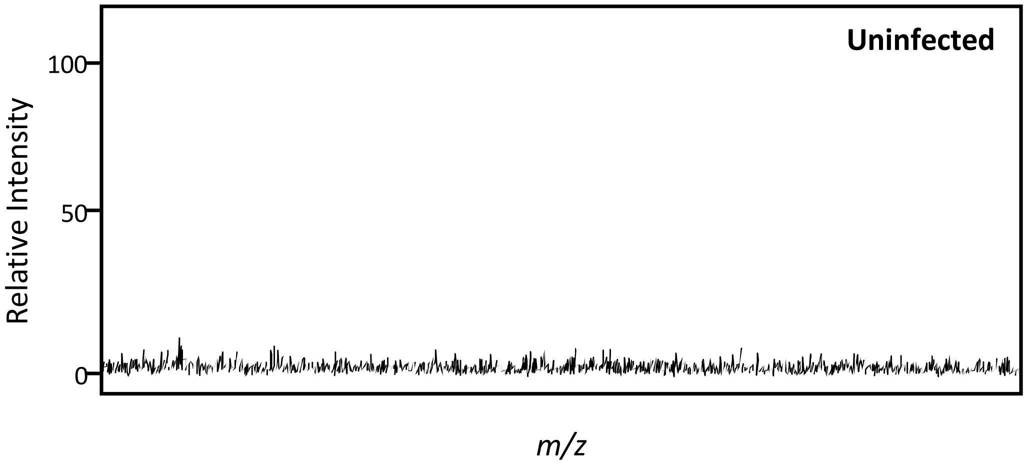

Figure S7. Mice underwent intranasal instillation with 50µl sterile PBS, serving as the mock-infected cohort for data shown in **Figure 4A.** Lipid A extraction of lung lavage fluid revealed no detectable lipid A.

Table S5. Primer sequences for qRT-PCR.

gene	sequence (5'-->3')
pagP_FWD	TTTCGACAGTGACAGCTACC
pagP_REV	GGGATCTTGTCGCGGTATT
pagL_FWD	CGACTCGATCAAGCCATTCA
pagL_REV	CTTCGAAGTTCAGGGAGGAAC
lpxO1_FWD	CTGATGTACCTGTTCTCGAAGG
lpxO1_REV	GTTCGGAACGCTTGATGTTG
lpxO2_FWD	GCGTCCTGTTCGTCCATTTC
lpxO2_REV	CAGGTACATCAGCGAGTTGTAG
rpsL_FWD	AAC TCG GCA CTG CGT AAG
rpsL_REV	TGTGCTCTTGCAGGTTGT
lptD_FWD	TCCTCGGGAAAAACTGACCG
lptD_REV	CCTTGGCGGATACGTAGGTC
oprD_FWD	CGCTACGCAATCACCGATAA
oprD_REV	GGGATGGTGTAGTTGCTGTT
oprF_FWD	ATCAAGAACCTGGCTGACTTC
oprF_REV	CGGACAGCTTCTGGTTGTAA

Table S6. Non-redundant bacterial LpxO sequences from NCBI nr protein database.

SI References

1. Chandler CE, Hofstaedter CE, Hazen TH, Rasko DA, Ernst RK. 2023. Genomic and Functional Characterization of Longitudinal Pseudomonas aeruginosa Isolates from Young Patients with Cystic Fibrosis. Microbiol Spectr https://doi.org/10.1128/spectrum.01556-23.

2. Edgar RC. MUSCLE: multiple sequence alignment with high accuracy and high throughput. *Nucleic Acids Res*. 2004;32(5):1792-1797. Published 2004 Mar 19. doi:10.1093/nar/gkh340

3. Crooks GE, Hon G, Chandonia JM, Brenner SE. WebLogo: a sequence logo generator. Genome Res. 2004;14(6):1188-1190. doi:10.1101/gr.849004

4. Stamatakis A. RAxML version 8: a tool for phylogenetic analysis and post-analysis of large phylogenies. *Bioinformatics*. 2014;30(9):1312-1313. doi:10.1093/bioinformatics/btu033

5. Winsor GL, Griffiths EJ, Lo R, Dhillon BK, Shay JA, Brinkman FS. Enhanced annotations and features for comparing thousands of Pseudomonas genomes in the Pseudomonas genome database. Nucleic Acids Res. 2016;44(D1):D646-D653. doi:10.1093/nar/gkv1227

6. Cain, A. K., Nolan, L. M., Sullivan, G. J., Whitchurch, C. B., Filloux, A., & Parkhill, J. (2019). Complete genome sequence of pseudomonas aeruginosa reference strain Pak. *Microbiology Resource Announcements*, *8*(41). https://doi.org/10.1128/mra.00865-19

7. Jorgensen JH, Turnidge JD. Susceptibility test methods: dilution and disk diffusion methods. In: *Manual of Clinical Microbiology*. 9th Edition. ASM Press; 2007:1152-1172.

8. Miller AK, Brannon MK, Stevens L, et al. PhoQ mutations promote lipid A modification and polymyxin resistance of Pseudomonas aeruginosa found in colistin-treated cystic fibrosis patients. *Antimicrob Agents Chemother*. 2011;55(12):5761-5769. doi:10.1128/AAC.05391-11
